# Molecular Structure Effect on the Epoxidation of 1-Butene and Isobutene on the Titanium Silicate Catalyst under Transient Conditions in a Trickle Bed Reactor

**DOI:** 10.1021/acsomega.3c00087

**Published:** 2023-07-13

**Authors:** Matias Alvear, Marie-Louis Reich, Kari Eränen, Stefan Haase, Dmitry Yu. Murzin, Tapio Salmi

**Affiliations:** † Laboratory of Industrial Chemistry and Reaction Engineering (TKR), Johan Gadolin Process Chemistry Centre (PCC), Åbo Akademi University, Turku/Åbo, Finland; ‡ Chemische Verfahrens- und Anlagentechnik, Institut für Verfahrens- und Umwelttechnik, Technische Universität Dresden (TUD), Dresden, Germany

## Abstract

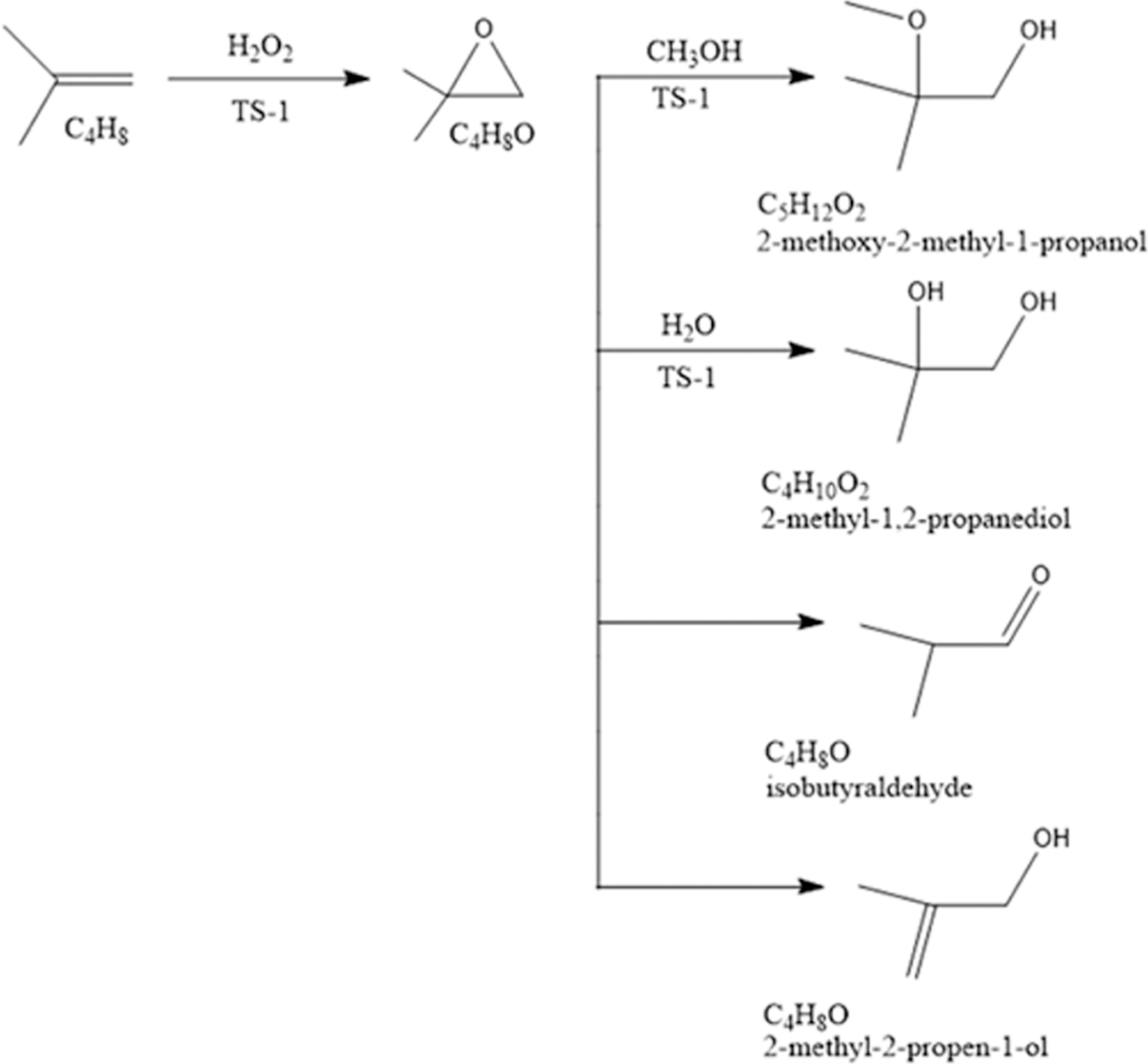

Epoxidation of two butane isomers (1-butene and isobutene) on the commercial titanium silicate (TS-1) catalyst was studied in a laboratory-scale trickle bed reactor. The transient step response technique was used as the main tool in the investigation. The transient responses revealed different dynamics of product formation in continuous operation. The study of isomers showed the impact of the molecular structure on the transient and stationary states of the system. The four-carbon chain present in 1-butene displayed a dynamic behavior with a prominent maximum of the conversion as a function of time-on-stream. On the contrary, the behavior of isobutene was displayed to be closer to ethene and propene under similar conditions reaching a steady state after ca. 2 h. The structure of the epoxide was an important factor in order to achieve a high epoxide selectivity. In isobutene epoxidation, the primary product 1,2-epoxy-2-methylpropane was highly reactive, giving a spectrum of parallelly formed byproducts. Therefore, the selectivity of the epoxide from isobutene was limited to ca. 70%. In the epoxidation of 1-butene, 1,2-epoxybutane was displayed to be a highly stable product with a selectivity close to 99%. Based on the transient and stationary data, a reaction mechanism was proposed for the epoxidation and ring-opening reactions present in the system.

## 1 Introduction

Epoxides are important chemical intermediates for the production of antifreeze agents, polymers, adhesives, and coatings among other products, for that during the last four decades, the epoxidation of light olefins with hydrogen peroxide on the titanium silicate 1 (TS-1) catalyst has been studied intensively. Direct epoxidation of propene to propene oxide has achieved successful industrial implementations under mild reaction conditions.^1^ However, even if ethene^2−5^ and 1-butene^6−11^ epoxidation has attended recent research interest, most of the previous research has aimed to compare different catalysts more than to investigate how the epoxidation reaction and side reactions are affected by the different process parameters. The studies have in most cases been limited to the use of batch reactor technology or fixed beds operating under stationary conditions.

The available literature reports studies on catalyst modifications,^12−16^ solvent effects,^1,17^ and reactor^18−21^ configurations for propene epoxidation, while for butenes, most of the previous investigations have been devoted to catalyst modifications applied in batch reactors without any detailed studies on the product selectivity and reactant conversion.^6−11^ Nevertheless, the studies carried out in continuous mode for 1-butene epoxidation are reported over 10 h of reaction displaying a constant decrease in the catalyst activity with time-on-stream in each catalyst tested, evidently due to the reactant and product capture on the catalyst surface.^6,7^ Therefore, it is important to study these two reaction systems in a broad set of conditions to understand if isomers have similar reaction mechanisms and catalyst activities. On the other hand, it is necessary to work in a continuous regime due to the changes reported for 1-butene between 10 and 350 h.^6,7^ Isobutene epoxidation has not been reported extensively; the study of the epoxidation of isobutene has been reported once to compare the rate with other olefins^11^ without any deeper analysis of the byproducts.

In the present work, the epoxidation of 1-butene and isobutene with hydrogen peroxide on a commercial titanium silicate catalyst was investigated in a laboratory-scale trickle bed reactor operating under stationary and transient conditions within a wide range of experimental parameters, such as reaction temperature, alkene pressure, and hydrogen peroxide and water concentrations as well as liquid flow rate. The main goal of this research work was to gain new insights into catalyst durability and product distribution depending on the molecular structure of the olefins.

## 2 Experimental Section

### 2.1 Heterogeneous Catalyst

Commercial titanium silicalite (TS-1) of ACS (Advance Chemical Supplier) material type B was the catalyst employed: CAS No. 13463-67-7 (titanium dioxide)/7621-86-9 (silicon dioxide); the microporous Ti–Si molecular sieve was prepared by a hydrothermal method.^22^ The surface area, pore size distribution, and pore volume of the catalyst material were measured with nitrogen physisorption using Micromeritics 3 Flex equipment. The results were interpreted with the Dubinin–Radushkevich and density functional theory (DFT) methods. The catalyst sample was degassed two times before the measurement: first ex situ for a period of 24 h at 180 °C and 0.1 mbar, followed by in situ degassing at 180 °C and 0.05 mbar.

### 2.2 Chemicals

The gases used were helium with 1 mol % nitrogen (AGA), 1-butene (AGA), and isobutene (AGA). The liquids utilized were aqueous hydrogen peroxide solution (>30 w/v%, Fisher Chemicals), methanol (>99.9%, Honeywell), 1,2-epoxybutane (99%, Sigma-Aldrich), 1-methoxy-2-butanol (97%, Sigma-Aldrich), 1,2-epoxy-2-methylpropane (97%, Sigma-Aldrich), isobutyraldehyde (99,9%, Sigma-Aldrich), 2-methyl-2-propen-1-ol (98%, Sigma-Aldrich), 1-methoxy-2-methyl-2-propanol (99,9%, Sigma-Aldrich), and 2-methyl-1,2-propanediol (99.71%, BLDpharm). For titrimetric analysis, ferroin indicator (0.1 wt %, Sigma-Aldrich), cerium(IV) sulfate solution (0.1 M, Honeywell), and 1,2-butanediol (>98%, Sigma-Aldrich) were used. All of the chemicals were used without further purification.

### 2.3 Experimental Setup and Procedures

**Figure 1:**
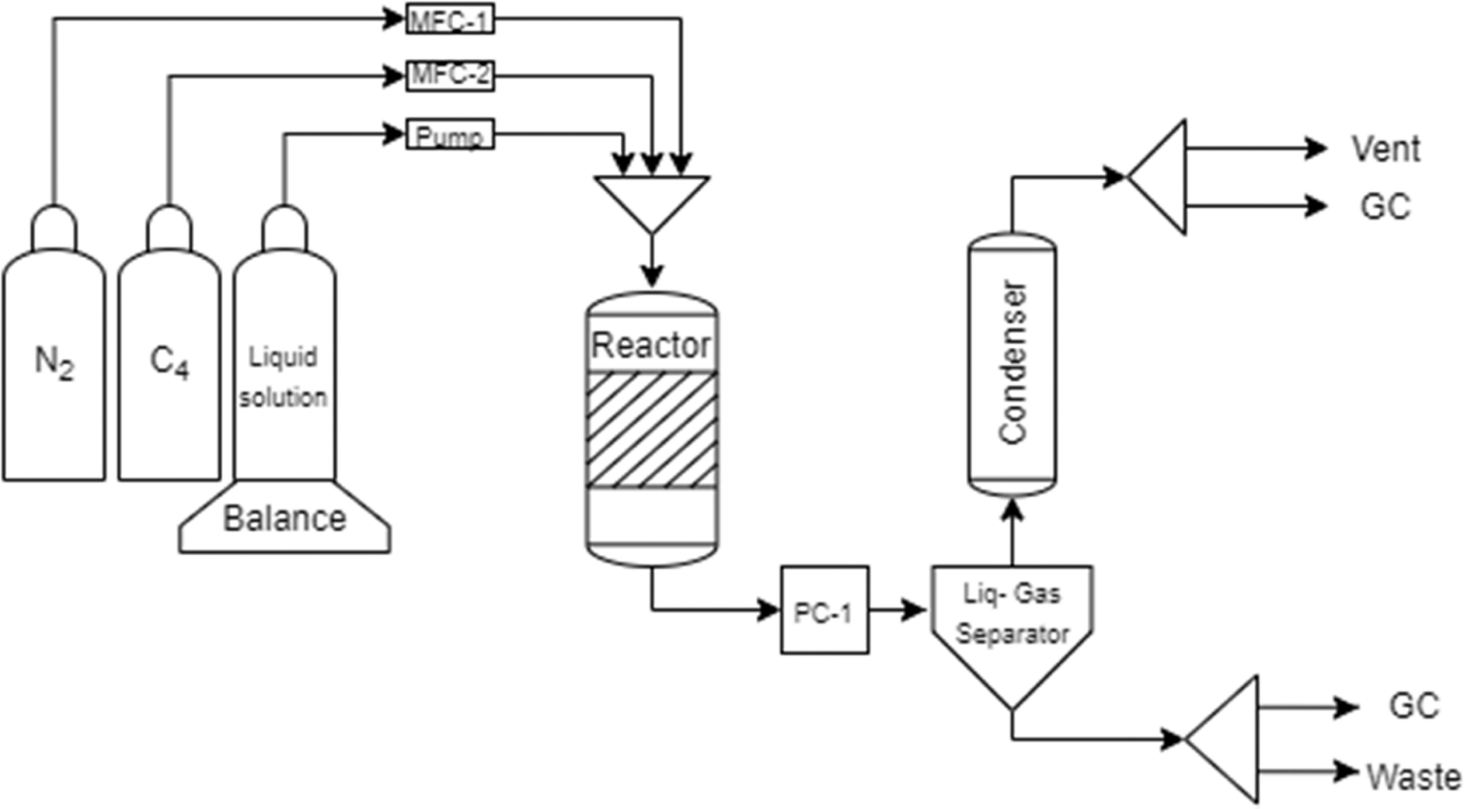
Experimental setup utilized for 1-butene and isobutene epoxidation in a laboratory-scale trickle bed reactor system.

The experimental work was carried out in the setup schematically illustrated in Figure 1. Hydrogen peroxide diluted in methanol was fed along with the gas phase containing 1-butene or isobutene across a packed bed reactor with 1.5 cm of internal diameter and 34 cm of length. The reactor tube was filled with 1 g of the TS-1 catalyst with particle sizes between 125 and 250 μm, which were mixed with 20 g of quartz beads. Small TS-1 catalyst particle sizes were used to suppress the internal mass transfer limitation in the catalyst pores.

The liquid phase was fed through an HPLC pump (Agilent 1100 Series), while the gas phase was fed into the system through mass flow controllers (Brooks Instruments). An Equilibar pressure controller (U3L Ultra Low Flow Back Pressure Regulator) was placed after the reactor outlet (PC-1), where the gas and liquid phases were separated in a 50 mL recipient operating under atmospheric conditions. The clogging of the gas chromatograph column was avoided with a condenser operating at 10 °C prior to the chromatograph.

### 2.4 Chemical Analysis

An Agilent gas chromatograph 6890N (G1540N) with a capillary column (Plot U and Molsieve) with a length of 60 m, a diameter of 530 μm, and an active-phase thickness of 20 μm was utilized to analyze the gas- and the liquid-phase samples from 1-butene epoxidation. In total, 1-butene and nitrogen were calibrated by sampling each gas several times. The calibration of the liquid compounds was done (1,2-epoxybutane, 1,2-butanediol, and 1-methoxy-2-butanol) with solutions in methanol (10, 5, 2.5, and 1.25 wt %).

The analysis of the gas-phase composition in isobutene epoxidation was performed with an Agilent 490 micro gas chromatograph equipped with a CP-Sil 5CB column with a length of 6 m, a diameter of 0.32 mm, and an active-phase thickness of 20 μm. For the liquid-phase analysis, an Agilent gas chromatograph 6890N (G1540N) was utilized. Nitrogen and isobutene were calibrated by sampling each gas 10 times. The calibration of the liquid phases was done with four samples of each compound (1,2-epoxy-2-methylpropane, isobutyraldehyde, 2-methyl-2-propen-1-ol, 1-methoxy-2-methyl-2-propanol, 2-methyl-1,2-propanediol) in methanol solutions (5, 2.5, 1.25, and 0.625 wt %).

Before each experiment, the hydrogen peroxide concentration was confirmed by titration with a cerium(IV) sulfate (Ce(SO_4_)_2_) solution. Ferroin was used as an indicator in the titrations.

### 2.5 Catalytic Experiments

The experimental activities of 1-butene and isobutene epoxidation comprised 22 and 23 experiments, respectively (Tables S2 and S3 in the Supporting Information). The reproducibility of the experiments was ensured with the repetition of three long-term (24 h) experiments. The effects of the liquid flow rate (0.5–3 mL/min), 1-butene and isobutene partial pressures (0.23–0.51 bar), water concentration (4.5–40 wt %) in methanol, hydrogen peroxide concentration (1–8 wt %), and temperature (15–50 °C) were studied. During all of the experiments, the same catalyst batch was used. The presence of trickle flow conditions was checked and based on flow charts for three-phase packed beds; it was confirmed that the reactor operated in the trickle flow mode.^20,22,23^ The experiments were carried out at 1 bar manometric (ca. 2 bar absolute pressure).

### 2.6 Calculation of Reactant Conversion and Product Yield and Selectivity

The alkene conversion was calculated as the total molar flow rate of consumption divided by the molar flow rate in the feed
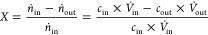
where *ṅ*_in_ and *ṅ*_out_ are the inlet and outlet molar flows, respectively, *c*_in_ and *c*_out_ are the inlet and outlet concentrations, respectively, and *V̇*_in_ and *V̇*_out_ are the inlet and outlet volumetric flows, respectively. The product selectivity was calculated as the total molar rate of production of the desired product divided by the total molar rate of consumed alkene.
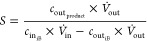


The product yield was defined according to
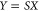


### 2.7 Blank Test and Reactor Cleaning

Prior to the kinetic studies, the reactor was filled with quartz sand to check the potential presence of chemical reactions in the absence of the catalyst. These experiments were carried out at 40 °C and 1 bar. The liquid flow contained 2 wt % hydrogen peroxide, water, and methanol, while the gas flow was 0.44 mmol/min nitrogen and 1-butene or isobutene mixed in an equimolar proportion. During this experiment, no products were detected, thus confirming the absence of noncatalytic reactions or reactions induced by the reactor wall.

After each experiment, the reactor column was fed for 1 h with a liquid phase consisting of methanol and a gas phase consisting of nitrogen. This was done in order to eliminate accumulated products from the catalyst pores and catalyst surface. The treatment temperature was 80 °C and the pressure was 4 bar.

## 3 Results and Discussion

### 3.1 Catalyst Characterization Results

The specific surface area of the commercial titanium silicalite (TS-1) catalyst was 450 m^2^/g, obtained with nitrogen physisorption and interpreted with the Dubinin–Radushkevich theory. The average pore size and the pore volume were 6.6 nm and 0.42 cm^3^/g, respectively.

### 3.2 Epoxidation Experiments

#### 3.2.1 Reaction Scheme and Catalyst Durability

**Scheme 1:**
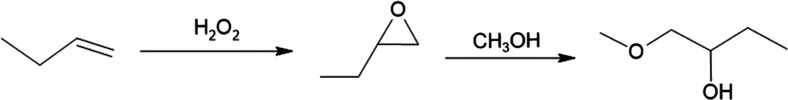
Proposed Simple Reaction Scheme for 1-Butene Epoxidation

Scheme 1 illustrates the overall reactions proposed previously for the epoxidation of 1-butene over the TS-1 catalyst.^6,7,11^ According to this scheme, 1-butene is transformed to 1,2-epoxybutene after which the epoxide can react further with methanol through ring opening and nucleophilic substitution to generate 1-methoxy-2-butanol as a secondary ring-opening product.

**Figure 2:**
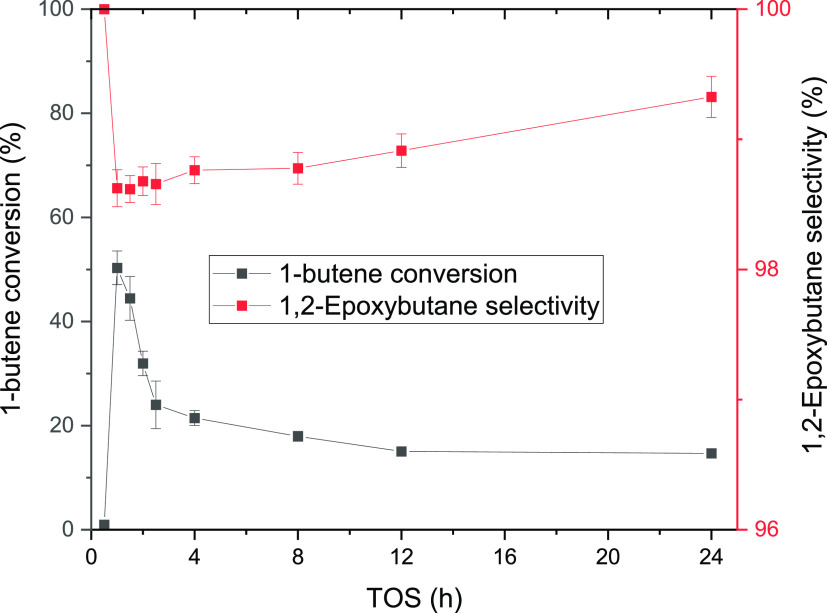
24 h experiments for 1-butene epoxidation conducted at 40 °C and 1 bar. 0.22 mmol/min of 1-butene (0.5 bar) was fed along with the liquid phase composed of 2 wt % H_2_O_2_ (0.24 mmol/min), 5 wt % H_2_O, and 93 wt % CH_3_OH. The liquid flow rate was 0.5 mL/min.

To further study the reaction scheme, the catalyst stability and selectivity were monitored with prolonged (24 h) experiments time-on-stream (TOS). The results are displayed in Figure 2. During the three successive experiments performed, changes in the catalyst activity with time-on-stream were noticed. At the start-up of the experiment, the 1-butene conversion increased reaching a maximum, after which it decreased. However, even if the activity change with time was observed, the excellent reproducibility of the experiments was confirmed as shown in Figure 2, and the catalyst was fully regenerated in the cleaning treatment (Section 2.7). Even though the 1-butene conversion declined, the selectivity of 1,2-epoxybutane was well preserved, the only byproduct being 1-methoxy-2-butanol, evidently formed from a reaction between methanol and 1,2-epoxybutane. Nonetheless, it is important to recognize the possibility of traces of 2-methoxy-1-butanol generated during the reaction because of the equilibrium between the methoxy species. However, because of the high selectivity to the epoxide 1,2-epoxybutene, no 2-methoxy-1-butanol was detected in the gas chromatographic analysis. The standard deviation in the conversion measurements was higher during the first 4 h and it was defined to be less than 5% after that period, whereas for the selectivity results, the deviations were less than 0.1%. The TOS behavior of 1-butene displayed to be different from ethene^22^ and propene^20^ epoxidation. In ethene and propene, the steady state was reached after 2.5 h.^20,22^ However, 1-butene reached stability after 12 h, almost five times longer than shorter olefins. The epoxide selectivity for ethene and propene was stable, around 90%, while for 1-butene, the selectivity increased with time-on-stream (Figure 2). Nevertheless, the selectivity always exceeded 98%.

Previously, this system has been reported from 10 h to 350 h.^6,7^ This is the first time when the first 10 h are reported. With the addition of this interval, it is possible to observe a correlation between the increase in the product selectivity and the decrease of the catalyst activity,^6,7^ because, as it is described in the literature, the decrease in the conversion can be correlated to the higher concentration of byproducts during the start of the reactor.

The 1-butene system displayed a different behavior compared with the epoxidation of propene carried out previously by us under similar conditions:^20^ the transient response of the propene oxide increased monotonically during the experiment. The reason for the behavior of 1,2-epoxybutane response with time-on-stream might be the capture of reactants and products in the catalyst structure, which was confirmed by the high amounts of reactants and products identified during the flushing of the reactor with methanol after each experiment (Section 2.7).

**Scheme 2:**
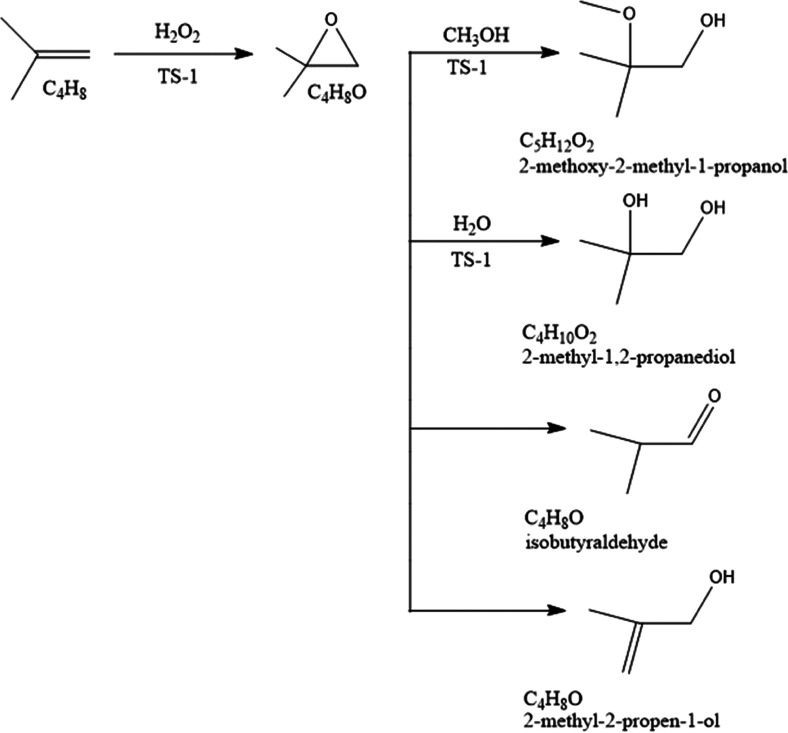
Confirmed Overall Reaction Scheme for Isobutene Epoxidation

In the case of isobutene, the epoxidation process resulted in highly reactive 1,2-epoxy-2-methylpropane and various secondary byproducts. In addition to the main reaction, ring-opening reactions with water and methanol took place, as confirmed by gas chromatography, leading to the secondary products isobutyraldehyde, 2-methyl-2-propen-1-ol, 1-methoxy-2-methyl-2-propanol, 2-methoxy-2-methyl-1-propanol, and 2-methyl-1,2-propanediol. The results suggest a consecutive-parallel stoichiometric pattern as displayed in Scheme 2.

**Figure 3:**
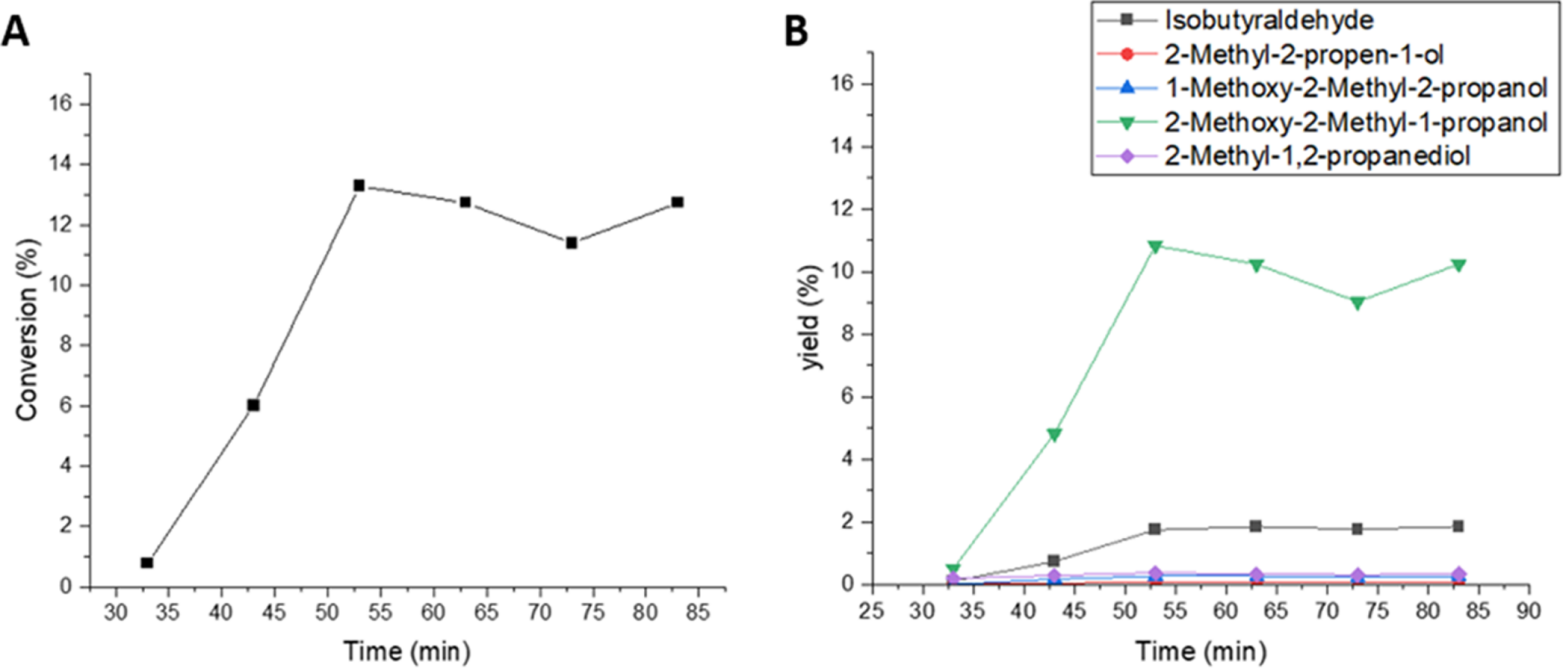
Ring-opening experiment. Conversion of 1,2-epoxy-2-methylpropane (A) and byproduct yields (B) in the epoxidation of isobutene. Conducted at 40 °C and 1 bar. 0.44 mmol/min nitrogen was fed along with the liquid phase composed of 2 wt % 1,2-epoxy-2-methylpropane in methanol. The liquid flow rate was 0.5 mL/min.

**Figure 4:**
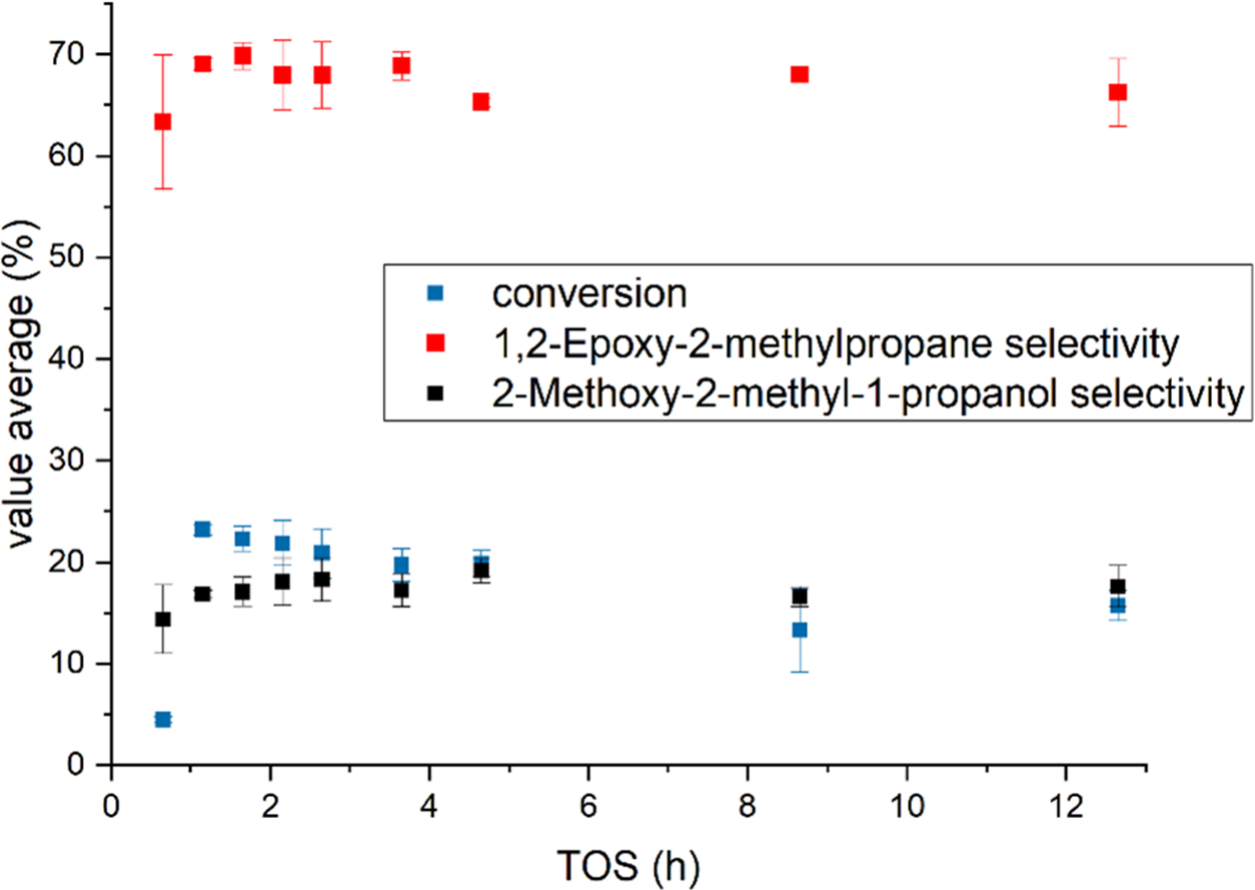
12 h experiments for isobutene epoxidation conducted at 15 °C and 1 bar. 0.40 mmol/min 1-butene (0.5 bar) was fed along with the liquid phase composed of 2 wt % H_2_O_2_ (0.24 mmol/min), 5 wt % H_2_O, and 93 wt % CH_3_OH. The liquid flow rate was 0.5 mL/min.

The important issue is whether the secondary products displayed in Scheme 2 are formed from the epoxide or not. Therefore, an additional experiment was carried out to confirm the reaction scheme. Figure 3B shows the secondary products as a function of time and Figure 3A represents the achieved conversions. The results indicate that all of the secondary products are formed directly from the epoxide and that the reaction network for the epoxidation of isobutene (Scheme 2) is valid. The catalyst stability for the isobutene epoxidation was investigated by three 12 h time-on-stream (TOS) experiments conducted at 1 bar. Figure 4 represents the selectivity of 1,2-epoxy-2-methylpropane and 2-methoxy-2-methyl-1-propanol as well as the conversion over 12 h time-on-stream.

The selectivity of the epoxy species slightly decreases with higher TOS with an average of 67% and a standard deviation of 2.7%. Furthermore, the 1-methoxy-2-methyl-2-propanol selectivity was equal to 17%. On one hand, the results demonstrate good catalyst stability and experimental repeatability over 113 h. On the other hand, the differences in behavior between 1-butene (Figure 2) and isobutene (Figure 4) are clear. Although 1-butene is more selective, it is less stable with time-on-stream. The comparison of isobutene with ethene^22^ and propene^20^ indicates similar times to reach the steady state. Nonetheless, the selectivity of isobutene epoxidation is clearly lower, reaching stability at 67%. These results are a clear signal of how the molecular structure displays an important element for determining the dynamic behavior of the system and the production of byproducts.

#### 3.2.2 Temperature Effect

**Figure 5:**
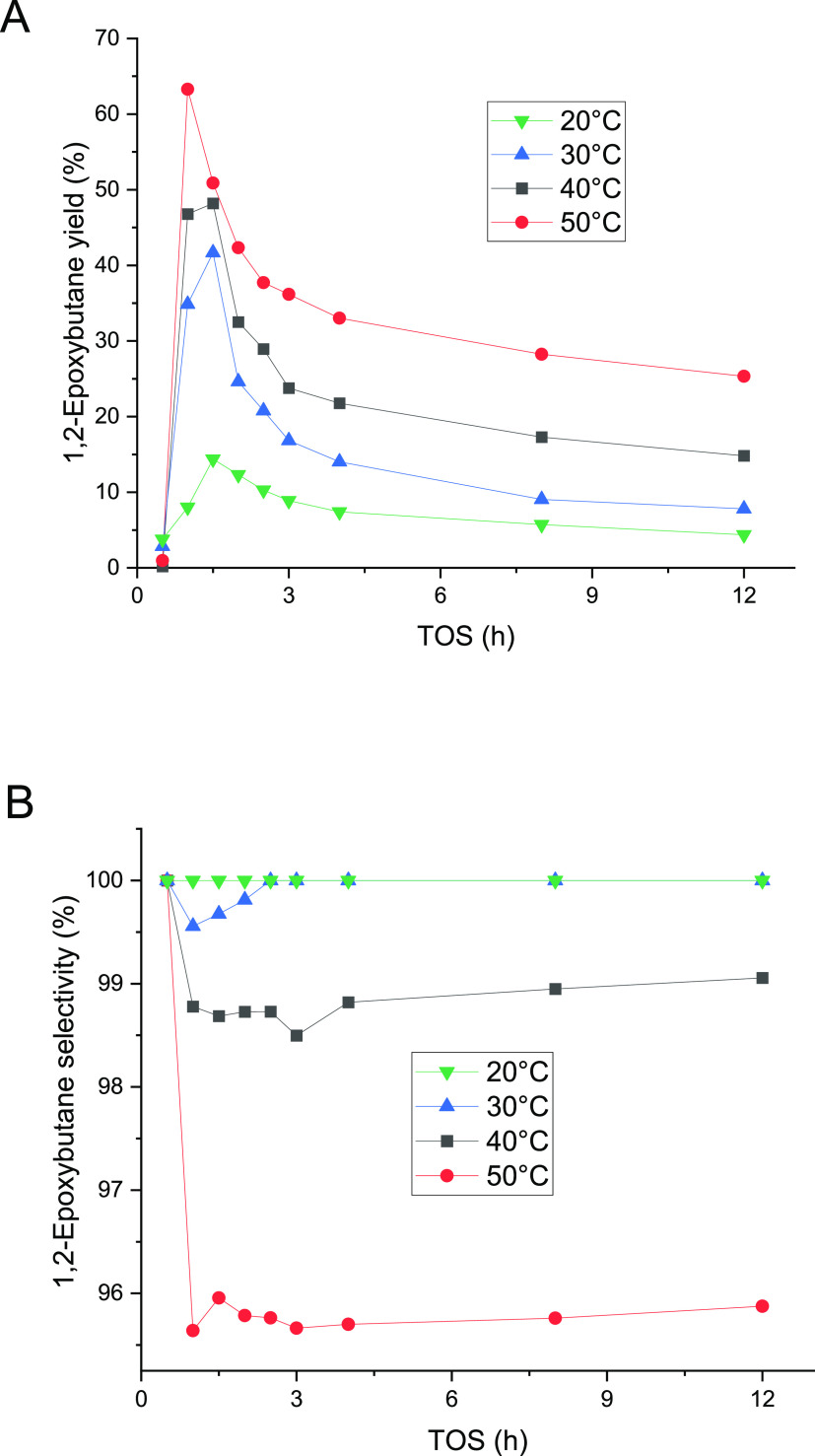
Temperature effect on the 1,2-epoxybutane yield (a) and selectivity (b) at 1 bar. The 1-butene feed was 0.22 mmol/min (0.5 bar) and the liquid phase comprised 2 wt % H_2_O_2_ (0.24 mmol/min), 5 wt % H_2_O, and 93 wt % of methanol, which were fed to the reactor. The liquid flow rate was 0.5 mL/min.

The temperature effect on reactant conversion and product distribution was studied. The increase of the reaction temperature had a positive influence on the reaction rate; however, the epoxide selectivity started to decrease slightly at temperatures above 30 °C as illustrated in Figure 5. All of the experiments displayed a very similar behavior with the time-on-steam. Nevertheless, the effect of the temperature on the 1,2-epoxybutane selectivity was not prominent in comparison with the propene epoxidation, where the selectivity was ca. 75% at 50 °C.^20,24^ For batch experiments for 1-butene epoxidation under similar conditions, selectivities to 1,2-epoxybutane of ca. 97% at 50 °C were reported.^9,10^ Higher temperatures than 50 °C were not studied here because our previous work confirmed that hydrogen peroxide decomposition on TS-1 played a role if the temperature exceeded 60 °C.^20^ The temperature can be considered an important variable in the production of the methoxy species: the increase in the production of byproducts with the increase of temperature indicates that the ring-opening process has a higher activation energy than the epoxidation reaction.

**Figure 6:**
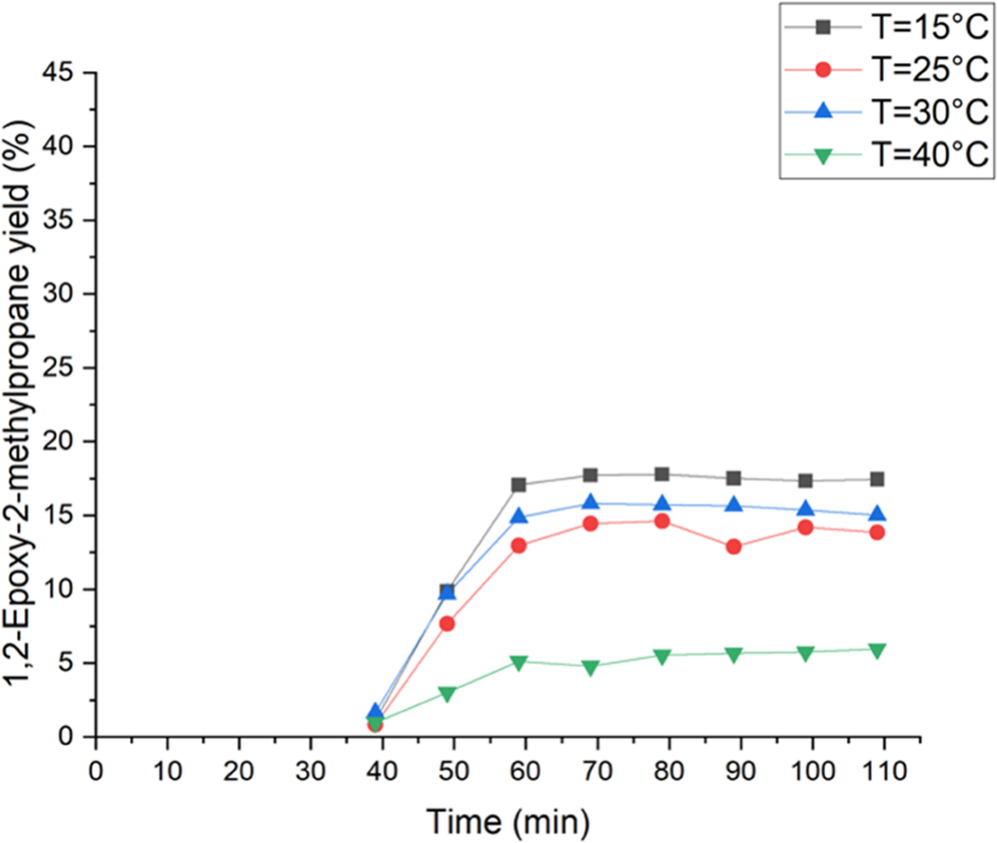
Temperature effect on the 1,2-epoxy-2-methylpropane yield. The isobutene feed was 0.40 mmol/min (0.5 bar) and the liquid phase comprised 2 wt % H_2_O_2_ (0.24 mmol/min), 5 wt % H_2_O, and 93 wt % of methanol, which were fed to the reactor. The liquid flow rate was 0.5 mL/min.

**Figure 7:**
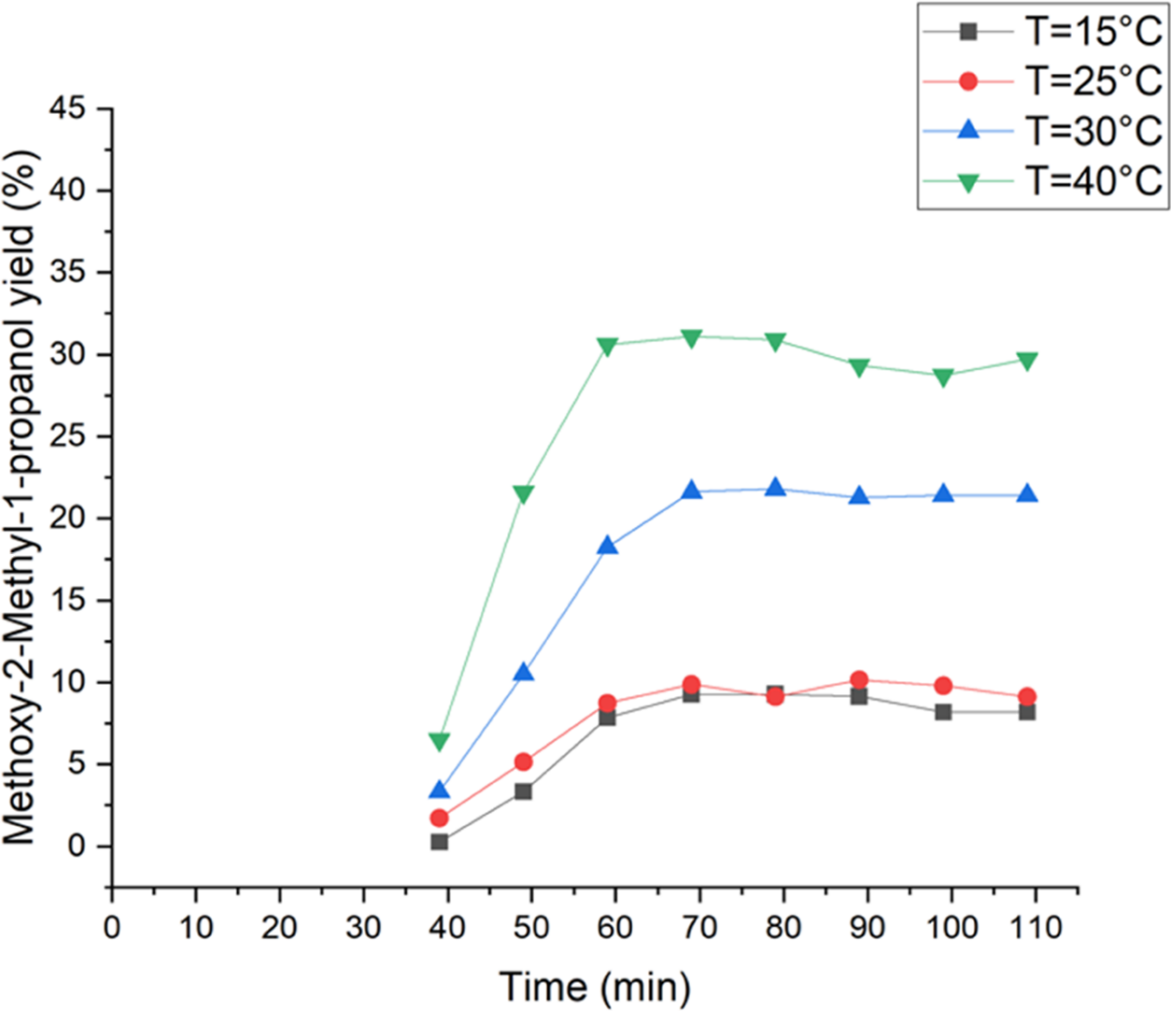
Temperature effect on the methoxy-2-methyl-1-propanol yield. The isobutene feed was 0.40 mmol/min (0.5 bar) and the liquid phase comprised 2 wt % H_2_O_2_ (0.24 mmol/min), 5 wt % H_2_O, and 93 wt % of methanol, which were fed to the reactor. The liquid flow rate was 0.5 mL/min.

The influence of the reaction temperature on the epoxidation of isobutene was investigated between 15 and 40 °C. The obtained results are presented in Figures 6 and 7. Table 1 shows the yields of all ring-opening byproducts, the epoxy species, and the conversions at different temperatures. Figure 6 shows the effect of the temperature on the 1,2-epoxy-2-methylpropane yield. The highest epoxide yield was detected at 15 °C. Furthermore, the yield steadily decreased with the increase of the temperature, while the conversion increased, as shown in Table 1. Especially at temperatures between 30 and 40 °C, the yield decreased rapidly. Compared to this, the yield of the dominant byproduct constantly increased with increasing temperature. Figure 7 illustrates the effect of the temperature on the methoxy-2-methyl-1-propanol yield. Consequently, the lowest yield was detected at 15 °C and increased at higher temperatures, especially between 25 and 40 °C from 10% to 30%. As observed for ethene epoxidation with hydrogen peroxide, the results suggest that the activation energy could have an impact on the formation of 2-methoxy-2-methyl-1-propanol.^22,25^ The formation of this dominant byproduct has evidently higher activation energy than isobutene epoxidation with hydrogen peroxide. In conclusion, it is better to conduct the epoxidation of isobutene under lower temperatures to obtain a higher yield of the epoxy species. Therefore, during this work, the isobutene experiments were carried out at 15 °C.

**Table 1:** Yields and Conversions in the Epoxidation of Isobutane: Temperature Effect

		experiment			
temperature (°C)		15	25	30	40
conversion (%)		28.6	26.0	44.7	41.3
yield (%)	1,2-epoxy-methylpropane	17.5	13.9	15.0	5.95
	isobutyraldehyde	2.0	1.9	2.0	1.6
	2-methyl-2-propen-1-ol	0.07	0.26	0.14	0.11
	1-methoxy-2-methyl-2-propanol	0.25	0.26	0.73	0.72
	2-methoxy-2-methyl-1-propanol	8.2	9.8	21.4	29.7
	2-methyl-1,2-propanediol	0.6	1.4	5.4	3.3

#### 3.2.3 Effect of Partial Pressure

**Figure 8:**
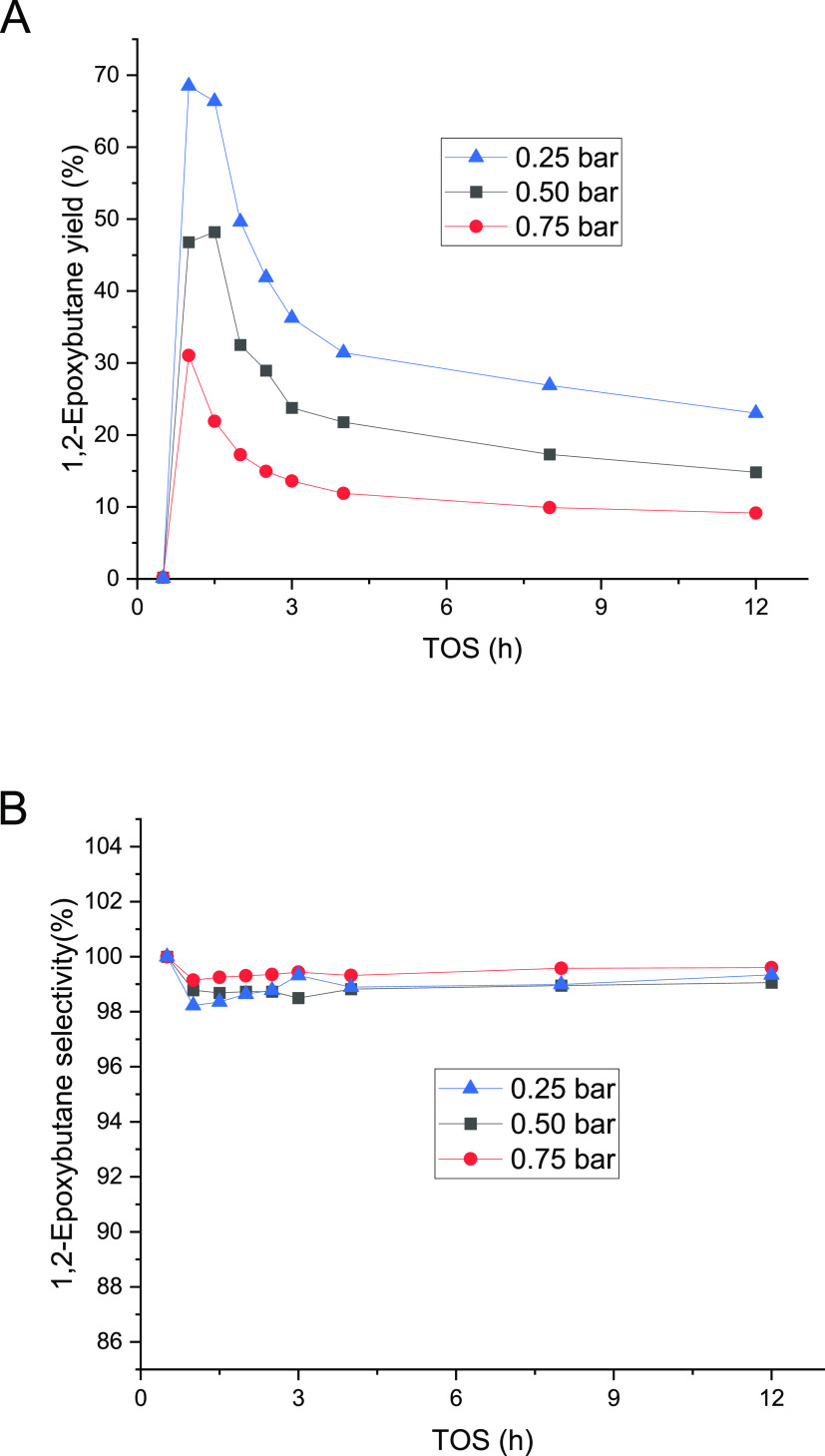
Effect of 1-butene partial pressure on the 1,2-epoxybutane yield (a) and selectivity (b) at 40 °C and 1 bar of total pressure. 1-Butene was fed along a liquid phase comprising 2 wt % H_2_O_2_ (0.24 mmol/min), 5 wt % H_2_O, and 93 wt % of methanol. The liquid flow rate was 0.5 mL/min.

In the epoxidation of 1-butene, Figure 8 illustrates a similar behavior with time-on-stream in all of the partial pressures screened. The 1,2-epoxybutane yield decreased with the increase of the partial pressure of butene, which it is expected because of the increase of the olefin concentration in the gas phase (Figure 8A). Nevertheless, the partial pressure did not affect the epoxide selectivity, which was very high, more than 98%, and almost constant after the first hour, as displayed in Figure 8B.

The effect of partial pressure was similar to the results reported previously for propene epoxidation: the conversion of the alkene decreases with the increase of the partial pressure.^20^ However, 1-butene maintained the high epoxide selectivity at different partial pressures, which was not the case in propene epoxidation.^20,24^ Furthermore, the partial pressure cannot be defined as an important parameter to suppress the catalyst deactivation with time because every yield curve displayed a proportional behavior in deactivation with time.

**Figure 9:**
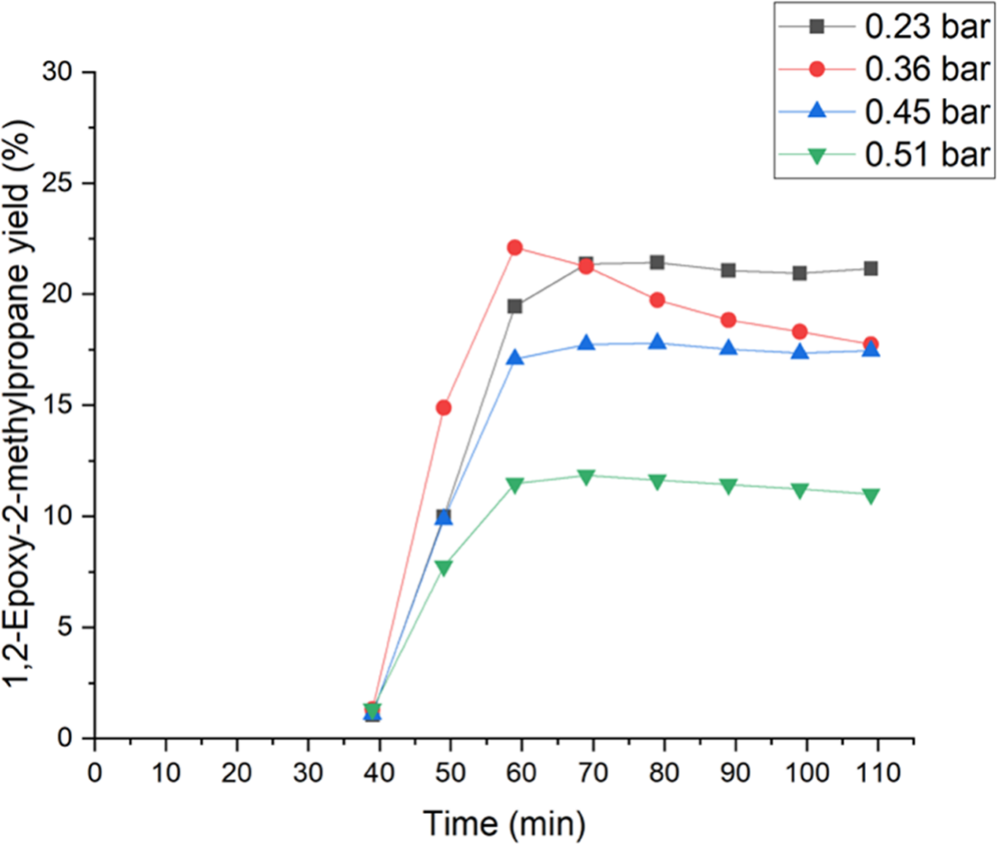
Influence of the isobutene pressure between 0.23 and 0.51 bar on the 1,2-epoxy-2-methylpropane yield at 15 °C and 1 bar of total pressure. The liquid phase comprised 2 wt % H_2_O_2_ (0.24 mmol/min), 5 wt % H_2_O, and 93 wt % methanol, which were fed to the reactor. The liquid flow rate was 0.5 mL/min.

**Figure 10:**
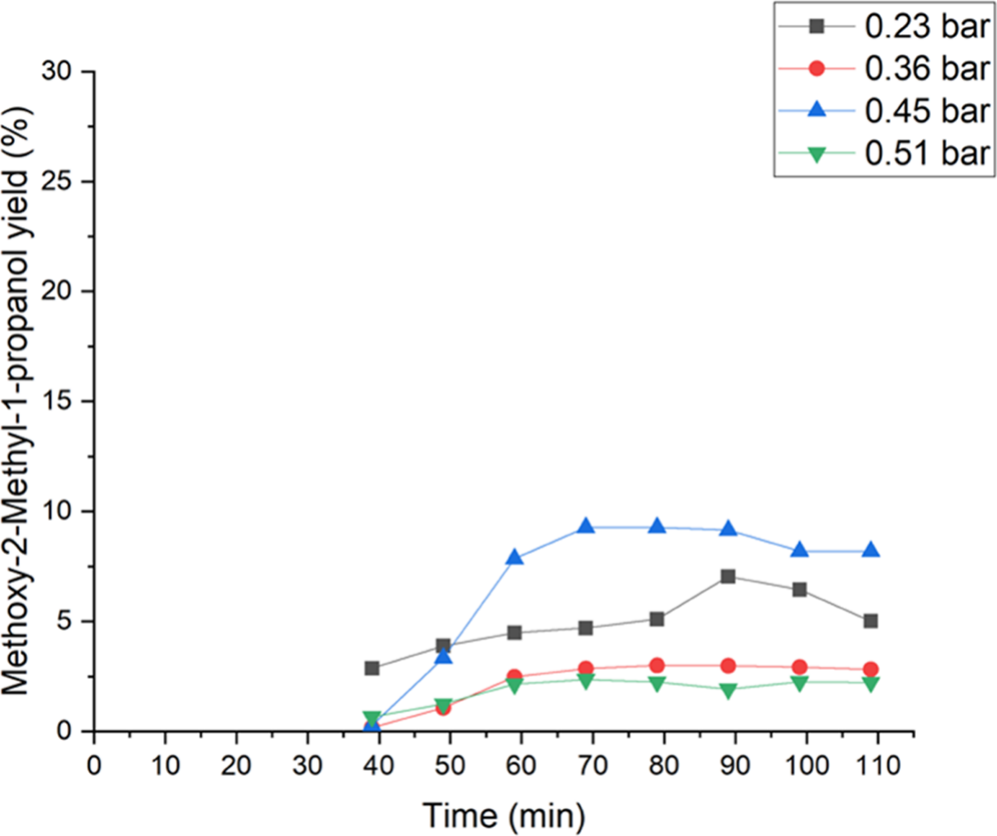
Effect of isobutene partial pressure on the 2-methoxy-2-methyl-1-propanol yield at different partial pressures between 0.23 and 0.51 bar. At 15 °C and 1 bar of total pressure. The liquid phase comprised 2 wt % H_2_O_2_ (0.24 mmol/min), 5 wt % H_2_O, and 93 wt % methanol, which were fed to the reactor. The liquid flow rate was 0.5 mL/min.

During the epoxidation of isobutene, the results were different. The yields of 1,2-epoxy-2-methylpropane as a function of the time are displayed in Figure 9. The results showed a negative effect on the 1,2-epoxy-2-methylpropane yield at higher isobutene partial pressures. Especially at partial pressures between 0.45 and 0.51 bar, the yield declines rapidly. A maximum was detected at 0.23 bar with 21.2%. Moreover, at 0.36 and 0.45 bar, the yields are slightly similar between 16.5 and 17.5%. Table 2 shows the conversions, the yields of the epoxy species, and the yields of the ring-opening products. The conversions and yields of the secondary products isobutyraldehyde and 2-methyl-2-propen-1-ol increased, while the isobutene partial pressure decreased. However, a constant conversion, which is independent of the isobutene pressure, could be observed. The yields of 2-methyl-2-propen-1-ol and 2-methyl-1,2-propane-diol were stable. Figure 10 shows the influence of different isobutene partial pressures on the 2-methoxy-2-methyl-1-propanol yield. A maximum was detected at 0.45 bar with 8.91%. Further, the yield slightly decreased at 0.23 bar. However, at 0.36 and 0.51 bar, the yields were low at 3.8 and 2.2%, respectively. Lower isobutene partial pressures prefer the formation of 1,2-epoxy-2-methylpropane (Table 3).

**Table 2:** Yields and Conversions in the Epoxidation of Isobutane: Partial Pressure Effect

		experiments			
partial pressure (bar)		0.23	0.36	0.45	0.51
conversion (%)		31.6	24.1	28.6	16.0
yield (%)	1,2-epoxy-methylpropane	21.2	16.6	17.5	11.0
	isobutyraldehyde	4.83	3.32	2.01	1.74
	2-methyl-2-propen-1-ol	0.19	0.21	0.18	0.05
	1-methoxy-2-methyl-2-propanol	0.27	0.15	0.2	0.7
	2-methoxy-2-methyl-1-propanol	5.01	3.76	8.91	2.22
	2-methyl-1,2-propanediol	0.11	0.06	0.58	0.28

**Table 3:** Yields and Conversions in the Epoxidation of Isobutane: Effect of Flow Rate

		experiment			
liquid flow rate (mL/min)		0.5	1	2	3
conversion (%)		28.6	24.3	26.8	27.7
yield (%)	1,2-epoxy-methylpropane	17.5	18.0	17.7	17.1
	isobutyraldehyde	2.01	3.15	4.24	5.02
	2-methyl-2-propen-1-ol	0.07	0.21	0.32	0.41
	1-methoxy-2-methyl-2-propanol	0.25	0.21	0.43	0.49
	2-methoxy-2-methyl-1-propanol	8.19	2.13	2.81	2.85
	2-methyl-1,2-propanediol	0.58	0.61	1.28	1.78

#### 3.2.4 Effect of Water Concentration

**Figure 11:**
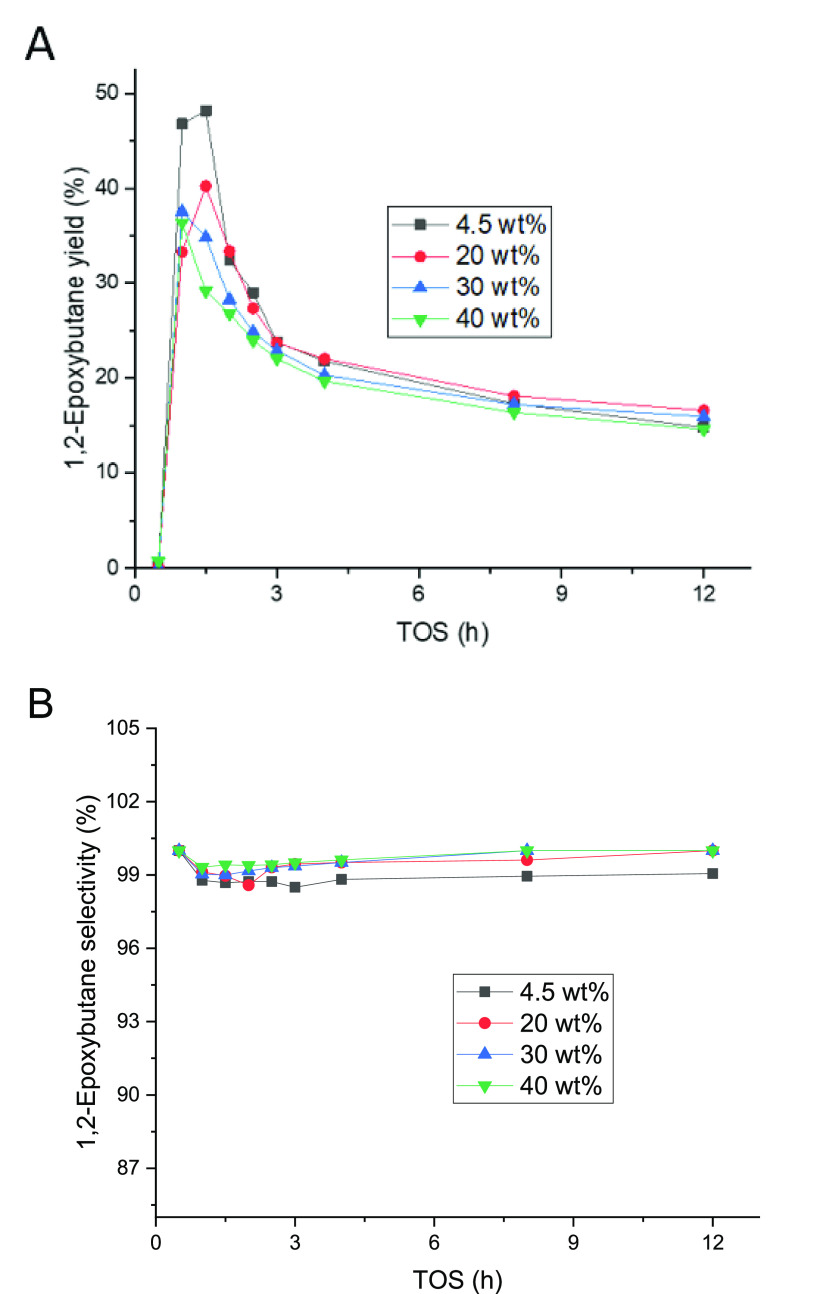
Water concentration effect on the 1,2-epoxybutane yield (a) and selectivity (b) at 1 bar and 40 °C. The 1-butene feed was 0.22 mmol/min (0.5 bar) and the liquid phase comprised 2 wt % H_2_O_2_ (0.24 mmol/min) and methanol, which were fed to the reactor. The liquid flow rate was 0.5 mL/min.

During 1-butene epoxidation, the results of modifying the water concentration were remarkably similar for every experiment (Figure 11). However, the epoxide selectivity improved slightly with the increase in water concentration. These results suggest a different behavior compared to propene epoxidation, where the presence of water induced important changes in the conversion and selectivity.^18,20,24^ The byproduct was 1-methoxy-2-butanol, while no 1,2-butanediol was observed during the experiments confirming that methanol but not water is the key nucleophile causing the ring opening of 1,2-epoxybutane.

**Figure 12:**
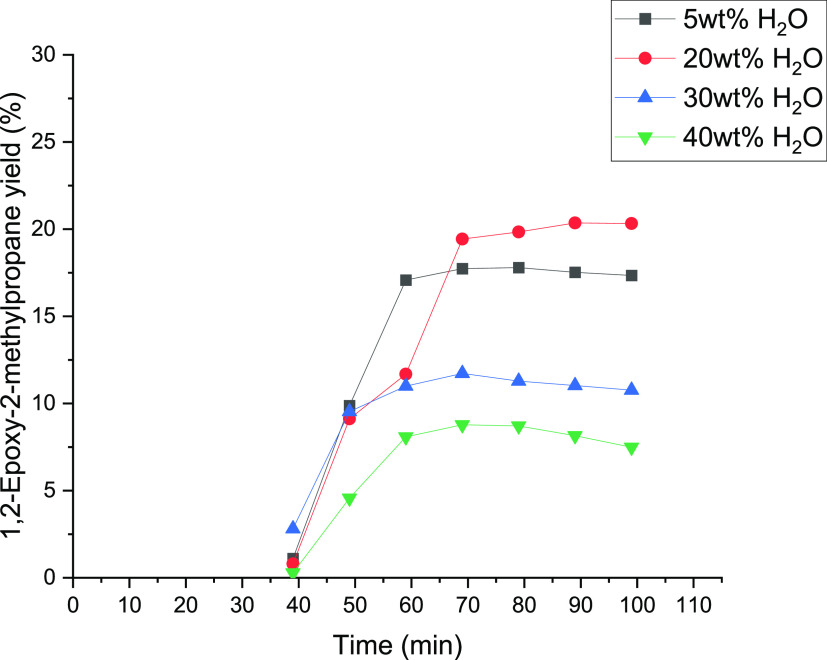
Influence of water concentrations of 5, 20, 30, and 40 wt % on the yield of 1,2-epoxy-2-methylpropane.

The effect of the water concentration was examined for isobutene too because water can adsorb on the catalyst surface, and through this, the water amount can influence the epoxidation kinetics.^22,25^Figure 12 shows the influence of the H_2_O concentrations on the yield of 1,2-epoxy-2-methylpropane. It displays an increasing yield between 5 and 20 wt%, whereby a maximum was observed at 20 wt %. However, at higher values of the water concentrations, the yield started to decrease. This might be explained by the decrease of the solubility of isobutene with increasing water amount (isobutene has higher solubility in methanol than that in water). In addition, with increasing water concentration, the solubility of 1,2-epoxy-2-methylpropane in the liquid phase increases, which could increase the ring-opening activity of 1,2-epoxy-2-methylpropane on the surface sites of the TS-1 catalyst.

**Figure 13:**
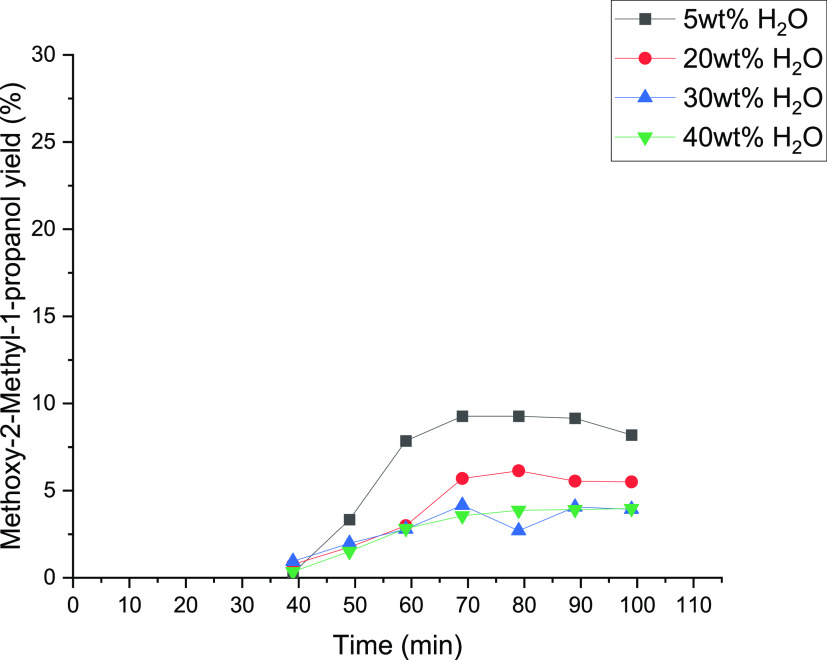
Influence of water concentrations of 5, 20, 30, and 40 wt % on the yield of methoxy-2-methyl-1-propanol.

At higher water concentrations, a decrease of isobutene conversion was observed. According to the literature, this might be related to the strong adsorption of water on the catalyst sites and the changes in the solubility of isobutene in the CH_3_OH/H_2_O_2_/H_2_O mixture.^24^ A maximum of conversion was detected between 5 and 20 wt %. Table 4 shows that an increasing amount of water modified the product distribution.^22^ On one hand, the yield of isobutyraldehyde, 2-methyl-2-propen-1-ol, and 2-methyl-1,2-propane-diol decreases, while the amount of water increases. Figure 13 represents the methoxy-2-methyl-1-propanol yield as a function of time. The yield shows the highest value of 8.2% at 5 wt % water concentration. An increase of the water concentration can lead to a reduction of the side products because of a lower total activity of isobutene on the TS-1 catalyst surface, as figured out in previous studies.^20,22^ Moreover, with the decrease of the water amount, the methanol concentration increases, which could support the formation of methoxy-2-methyl-1-propanol as the dominating byproduct. In conclusion, the results indicate the need to operate around 20 wt % to obtain the highest yield of the epoxy species. The differences with 1-butene epoxidation are observable. The concentration of water has an effect on the yield of isobutene epoxidation, while for 1-butene, the effect of the water concentration is minimal, as shown in Figure 11.

**Table 4:** Effect of the Water Concentration on Conversions and Yields

		experiment			
water concentration (wt%)		5	20	30	40
conversion (%)		28.6	29.7	19.3	17.6
yield (%)	1,2-epoxy-methylpropane	17.5	20.3	10.8	7.49
	isobutyraldehyde	2.01	1.64	2.03	2.92
	2-methyl-2-propen-1-ol	0.07	0.07	0.1	0.2
	1-methoxy-2-methyl-2-propanol	0.25	0.23	0.2	0.27
	2-methoxy-2-methyl-1-propanol	8.19	5.5	3.93	3.97
	2-methyl-1,2-propanediol	0.58	1.95	2.03	2.77

#### 3.2.5 Hydrogen Peroxide Effect

**Figure 14:**
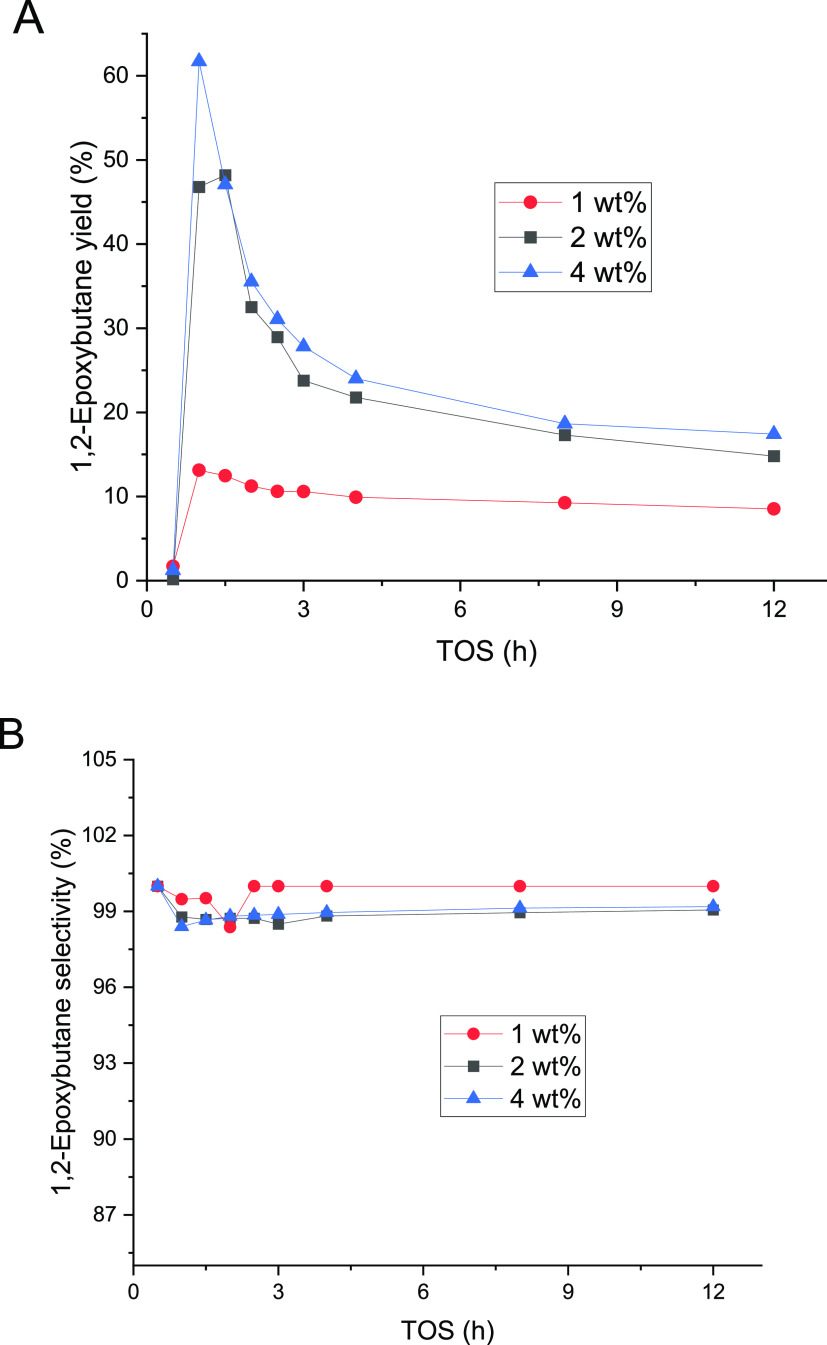
Hydrogen peroxide concentration effect on the 1,2-epoxybutane yield (a) and selectivity (b) at 1 bar. The 1-butene feed was 0.22 mmol/min (0.5 bar) and the liquid phase consisted of H_2_O_2_, H_2_O, and methanol, which were fed to the reactor. The liquid flow rate was 0.5 mL/min.

The experiments performed to study the hydrogen peroxide effect over the epoxidation of 1-butene displayed important differences between 1 and 2 wt % hydrogen peroxide; however, at concentrations exceeding 2 wt %, the differences were not significant, as shown in Figure 14. The epoxide selectivity behaved in the opposite way being highest at 1 wt % and decreasing at higher hydrogen peroxide concentrations. These results are similar to those observed for propene epoxidation, where the selectivity decreased between 1 and 4 wt % hydrogen peroxide concentrations.^20^

The transient behavior of the catalyst was displayed to be highly dependent on the amount of hydrogen peroxide (Figure 14a), 1 wt % being the best condition to start the operation of the reaction. This is because the low conversion decreases the amount of side products in the start-up of the system. Nevertheless, even if the activity is only half at 1 wt % compared to that at 2 or 4 wt % H_2_O_2_, the improvement in stability and selectivity over the entire experiment is demonstrated in Figure 14.

**Figure 15:**
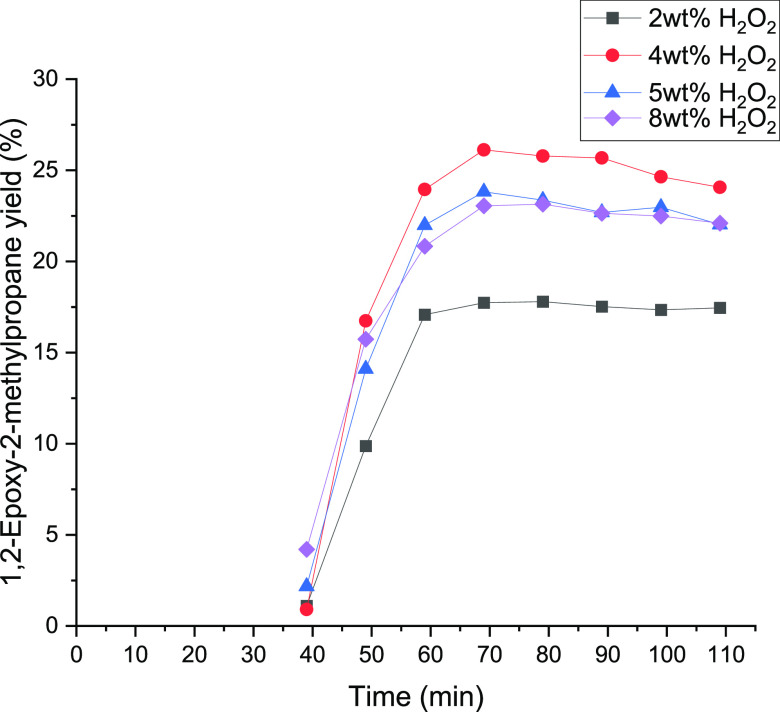
1,2-Epoxy-2-methylpropane yield as a function of time between 2 and 8 wt % hydrogen peroxide in solution.

**Figure 16:**
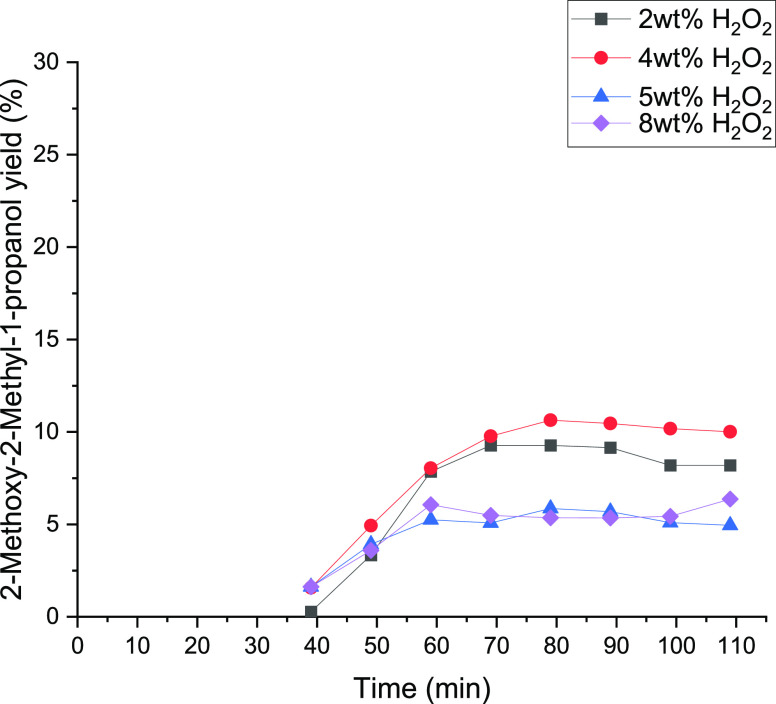
2-Methoxy-2-methyl-1-propanol yield as a function of time at 2, 4, 5, and 8 wt % hydrogen peroxide solutions.

The influence of the H_2_O_2_ concentration on the yield of 1,2-epoxy-2-methylpropane was studied too. The epoxidation of isobutene was carried out between 2 and 8 wt % H_2_O_2_. The used conditions are the same as those in the experiments representing the changes in the amount of water. The results are shown in Figures 15 and 16. Table 5 displays the summary of the conversions and the product yields at different hydrogen peroxide concentrations. A maximum yield of 1,2-epoxy-2-methylpropane of 24% was achieved at 4 wt % hydrogen peroxide. At 8 wt % H_2_O_2_, the yield of 1,2-epoxy-2-methylpropane was ca. 22%; this suggests a negative effect at high concentrations of hydrogen peroxide. However, the lowest yield was observed at 2 wt %. The decreasing yield could be explained by the reaction network of isobutene epoxidation in Scheme 2. With higher amounts of hydrogen peroxide, more isobutene can be converted to 1,2-epoxy-2-methylpropane. As shown in Table 5, the yield of isobutyraldehyde, 1-methoxy-2-methyl-2-propanol, and 2-methyl-2-propen-1-ol constantly increases with the increasing amount of hydrogen peroxide. In contrast to this, 2-methyl-1,2-propane-diol has two slightly similar maxima at 4 wt % and 8 wt %. The experimental data indicate that it is better to work at 4 wt % for maintaining a high epoxide yield.

**Table 5:** Effect of the H_2_O_2_ Concentration on Conversions and Yields

		experiment			
hydrogen peroxide concentration (wt %)		2	4	5	8
conversion (%)		28.6	39.7	31.1	36.9
yield (%)	1,2-epoxy-methylpropane	17.5	24.1	22.0	22.1
	isobutyraldehyde	2.01	2.17	2.99	4.91
	2-methyl-2-propen-1-ol	0.07	0.14	0.11	0.24
	1-methoxy-2-methyl-2-propanol	0.18	0.35	0.22	0.39
	2-methoxy-2-methyl-1-propanol	8.19	10.1	4.95	6.37
	2-methyl- 1,2-propanediol	0.58	2.86	0.76	2.92

Figure 16 displays the achieved yield of methoxy-2-methyl-1-propanol. The maximum was declared with the 4 wt % hydrogen peroxide solution. The yield as well as the amount of methanol decreases with increasing H_2_O_2_ concentration. Due to this, further ring-opening reactions with methanol on the catalyst surface get diminished. In addition, the water amount increases with higher hydrogen peroxide concentrations, which might cause a lower overall activity at the surface of the catalysts because of the decreasing solubility of isobutene in the CH_3_OH/H_2_O_2_/H_2_O mixture.^22^ Consequently, the minimum 2-methoxy-2-methyl-1-propanol yield was attained at 5 wt % with 4.95% and at 8 wt % with a similar value. In conclusion, working with a small excess of hydrogen peroxide is exhibited to be the best condition to perform the reaction with isobutene, while in the case of 1-butene, the best condition is working with an excess of olefin.

#### 3.2.6 Influence of the Liquid Flow Rate

**Figure 17:**
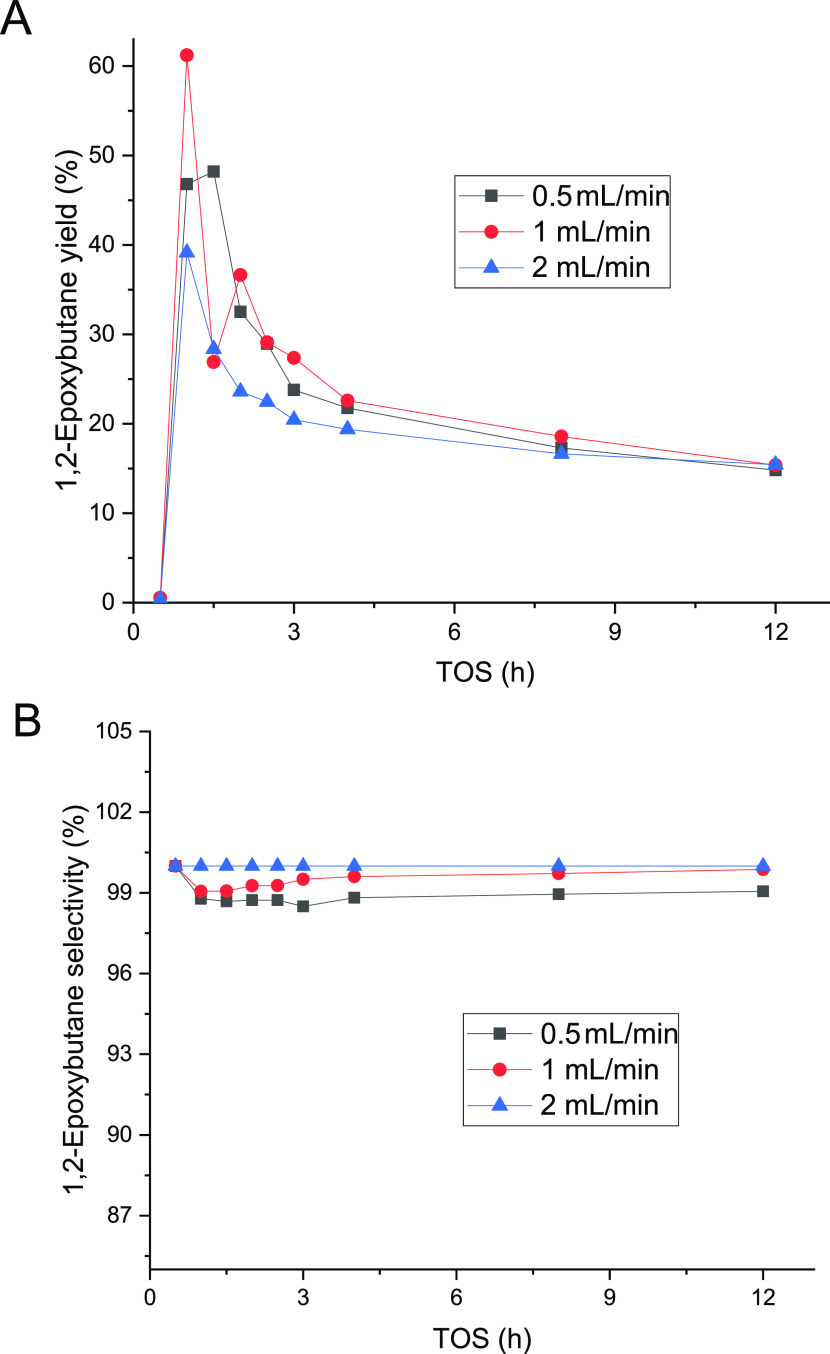
Liquid low rate effect on the 1,2-epoxybutane yield (a) and selectivity (b) at 1 bar and 40 °C. The 1-butene feed was 0.22 mmol/min (0.5 bar) and the liquid phase comprised 2 wt % H_2_O_2_, H_2_O, and methanol, which were fed to the reactor.

For the epoxidation of 1-butene, the liquid flow rate effect was investigated systematically at flow rates from 0.5 to 2 mL/min. The results are displayed in Figure 17. The liquid flow rates generated faster changes in the 1,2-epoxybutane yield with time-on-stream; however, it is possible to observe how all of the flows exhibited the same yield within 12 h. Nevertheless, the epoxide selectivity increased with the flow rate in the first few hours but at flow rates exceeding 1 mL/min, the selectivity was 100% at 12 h. The behavior of 1-butene with changes in the liquid flow is different from the conversions in propene epoxidation; however, the increase in the selectivity with time-on-stream was present in propene under similar conditions.^20^

**Figure 18:**
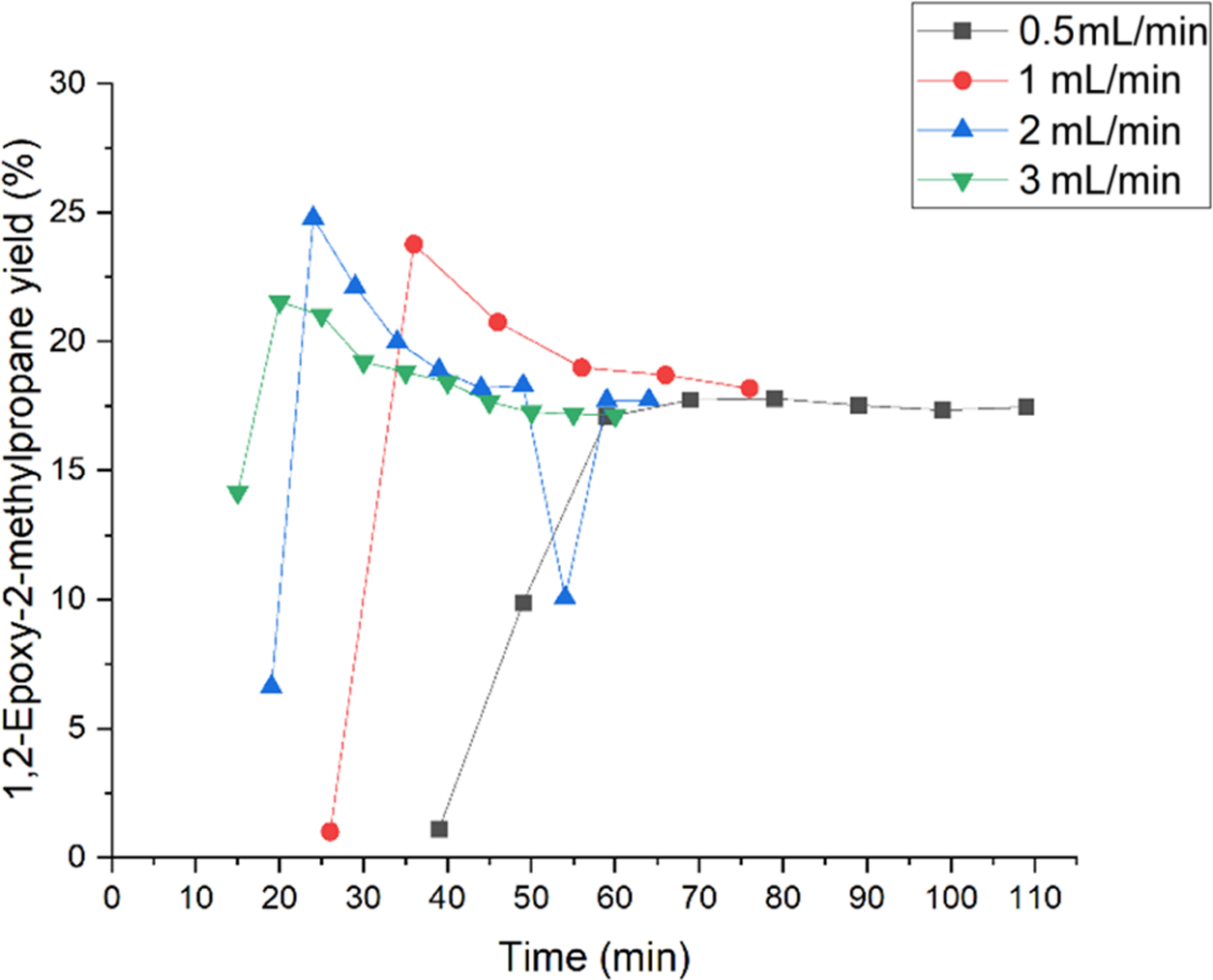
Influence of the flow rates of 0.5, 1, 2, and 3 mL/min on the 1,2-epoxy-2-methylpropane yield.

**Figure 19:**
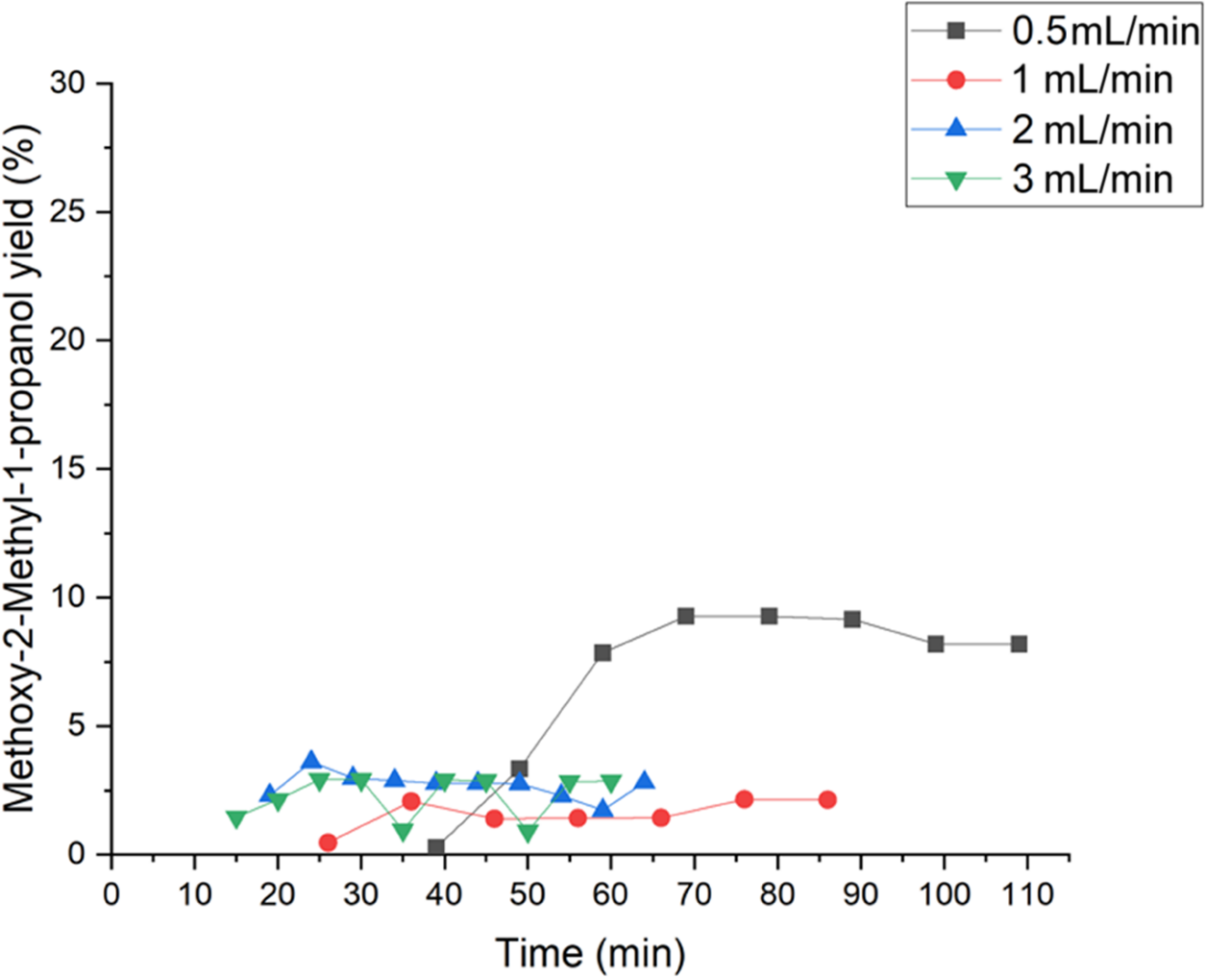
Influence of the flow rates of 0.5, 1, 2, and 3 mL/min on the methoxy-2-methyl-1-propanol yield.

In the case of isobutene, the liquid flow rates were studied at flow rates varying between 0.5 and 3 mL/min. The experimental results are presented in Figures 18 and 19. The experiments were continued until the steady state of the reaction system was attained. Figure 18 shows the yield of 1,2-epoxy-2-methylpropane as a function of time. The yields at steady state of all flow rates were detected to be rather similar between 17 and 18%. Table 3 provides the summary of the conversions and yields of all of the side products and the main product as well. A maximum of the conversion was identified at 0.5 mL/min with 28%. However, among the flow rates of 1 and 3 mL/min, the conversion slightly increases. The 2-methoxy-2-methyl-1-propanol yield is demonstrated in Figure 15. A maximum was detected at 0.5 mL/min with a value of 8.2%. Table 3 displays an increase of the 2-methyl-2-propen-1-ol, 1-methoxy-2-methyl-2-propanol, and 2-methyl-1,2-propane-diol yields with increasing liquid flows. Isobutyraldehyde yield increased with the increase of liquid flow and became the dominating byproduct. The increase of the byproducts at lower flow rates indicates the preferred formation of ring-opening products because of longer residence times in the reactor, resulting in longer contact times with the TS-1 catalyst.

### 3.4 Mechanistic Insights into 1-Butene and Isobutene Epoxidation and Ring-Opening Processes

**Scheme 3:**
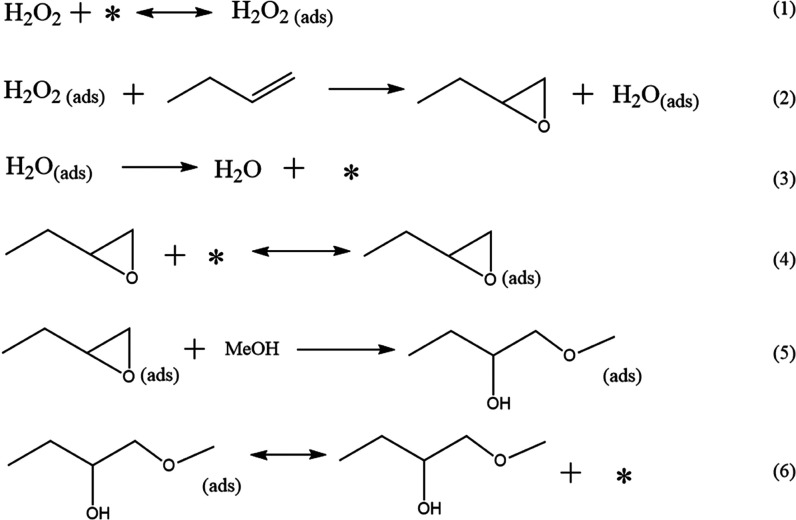
Proposed Alkene Epoxidation Mechanism on the TS-1 Catalyst

Although the selectivity to 1,2-epoxybutane was very high, exceeding 98% and even 99% in many experiments, the yield to 1,2-epoxybutane varied between 10 and 70%, depending on the temperature, 1-butene partial pressure, and hydrogen peroxide concentration. The mechanism for epoxidation of lower olefins has been described previously.^11,24,26−28^Scheme 1 displays the overall reaction stoichiometry of 1-butene if it would behave analogously with propene in the presence of TS-1. However, the results suggest that a simpler overall reaction scheme consisting of the formation of 1,2-epoxybutane and 1-methoxy-2-butanol is sufficient for the epoxidation of 1-butene. No 1,2-butanediol was detected in our experiments. Plausible consecutive epoxidation and ring-opening steps on the catalyst surface are displayed in Scheme 3.

Scheme 3 illustrates the reaction steps on the TS-1 surface according to the results observed in this work. The mechanism consists of four adsorption/desorption steps (1, 3, 4, and 6) and two surface reaction steps (2 and 5).

The reaction starts with the activation of the hydrogen peroxide on the titanium site.^28^ According to the literature, this step is facilitated by the solvent hydroxyl group.^26−28^ Step 2 describes the surface reaction between 1-butene and adsorbed hydrogen peroxide to produce 1,2-epoxybutane and water. Water stays attached to the catalyst surface until titanium silicalite is re-established through water desorption (step 3). The decline of the initial catalyst activity is related to the reactants and products remaining strongly adsorbed on the catalyst surface (steps 4–6). In step 4, 1,2-epoxybutane is adsorbed on the titanium site, activating the oxirane ring and facilitating the further reaction with methanol (step 5) to produce 1-methoxy-2-butanol as the ring-opening byproduct. Step 6 describes the adsorption/desorption of 1-methoxy-2-butanol. The 1-methoxy-2-butanol molecule is adsorbed strongly on the catalyst surface. However, it was possible to ensure the catalyst reusability and stability after flushing the reactor with methanol and nitrogen. Consequently, step 6 can be regarded as a reversible adsorption/desorption step.

The absence of appreciable changes in the catalyst behavior with the water increase is probably related to the lower activity displayed by the alkene reactant on the TS-1 catalyst because of the increase in the chain length and cross section^6,7,11^ compared to lower alkenes. The prominent influence of the hydrogen peroxide concentration on the epoxide yield suggests the importance of operating close to a stoichiometric ratio between 1-butene and hydrogen peroxide in order to get a high hydrogen peroxide efficiency because the reaction is in fact limited by the availability of dissolved 1-butene in the liquid phase.

In the case of isobutene, the system displays to behave partially in a similar way as that in the case of 1-butene. Nevertheless, the activity of epoxide is higher while reacting with four byproducts. The analysis of the experiments at different temperatures allowed us to plot the main byproduct concentration (methoxy-2-methyl-1-propanol) versus all of the other side products (Supporting Information S1). The results demonstrate linear plots, thus confirming the parallel formation of the secondary products directly from the epoxide. Therefore, Scheme 2 is valid as a representation of the reaction network.

## 4 Conclusions

Epoxidation of 1-butene and isobutene was studied in a broad range of experimental conditions (temperature, concentrations, flows, and partial pressures) on the commercial titanium silicalite (TS-1) catalyst. Transient and stationary experiments were conducted in the laboratory-scale trickle bed reactor in order to reveal catalyst durability and selectivity and to get new insights into the product formation and catalytic reaction mechanism.

The study of isomers of butene in the epoxidation on TS-1 revealed the importance of the molecular structure in the reaction system. The epoxidation of 1-butene was highly selective; however, the activity over time-on-stream changed. This behavior was not observed in ethene, propene, and isobutene, all of them with shorter carbon chains. The reactivity of the epoxide was highly affected by the molecular structure. The isobutene epoxidation displayed to be more prone to consecutive side reactions displaying lower selectivites of ca. 70%, while 1-butene exhibited a selectivity of 98% to the epoxide.

The epoxidation process in methanolic solvents usually produces methoxy and diol species as a result of ring opening. Nevertheless, the present study revealed new features in the epoxidation of isobutene. The second most common byproduct after the methoxy species was isobutyraldehyde. It is important to recall that aldehydes are not common products in the epoxidation of light linear olefins over the titanium silicate catalyst with hydrogen peroxide, and the separate experiment of 1,2-epoxy-2-methylpropane confirmed the aldehyde formation in an unequivocal way.

## Supporting Information Available

Product identification; concentration plots of isobutene epoxidation data; experimental matrix of 1-butene; and experimental matrix of isobutene (PDF)
