# Supplementary material for: Molecular Structure Effect on the Epoxidation of 1-Butene and Isobutene on the Titanium Silicate Catalyst under Transient Conditions in a Trickle Bed Reactor
Source: ACS Omega. 2023 Jul 13;8(29):25710–26. doi: 10.1021/acsomega.3c00087 (PMC10372949; doi:10.1021/acsomega.3c00087)
Supplement: Supplementary file 1 — ao3c00087_si_001.pdf [file ao3c00087_si_001.pdf]

Molecular structure effect on the epoxidation of 1-butene and iso-butene on titanium silicate catalyst under transient conditions in a trickle bed reactor

Matias Alvear[1], Marie-Louis Reich[1,2], Kari Eränen[1], Stefan Haase[2], Dmitry Yu. Murzin[1], Tapio Salmi[1]*

[1]Laboratory of Industrial Chemistry and Reaction Engineering (TKR), Johan Gadolin Process Chemistry Centre (PCC), Åbo Akademi University, Turku/Åbo Finland

[2]Chemische Verfahrens- und Anlagentechnik, Institut für Verfahrens- und Umwelttechnik, Technische Universität Dresden (TUD), Dresden, Germany

*tapio.salmi@abo.fi

Supporting Information

The present supporting information includes the product identification performed for iso-butene, the concentration plots of iso-butene epoxidation data for analyze the consecutive reactions and two tables listing the entire set of experiments performed in the publication.

Product identification

The various products which passed through the column were identified in a detector and recorded at a chromatogram. All in all, eight main peaks were seen at the chromatogram separated in Table S1.

Table S1. Components in the epoxidation of iso-butene identified by GC (RT= retention time/min)

RT	Identified compound	RT	Identified compound
11.299	iso-butene	20.265	2-methyl-2-propen-1-ol
11.672	methanol	21.501	1-methoxy-2-methyl-2-propanol

16.855	1,2-epoxy-2-methylpropane	22.765	2-methoxy-2-methyl-1-propanol
17.749	isobutyraldehyde	23.879	2-methyl-1,2-propanediol

Concentration plots of iso-butene epoxidation data

Concentrations as a function of time at different temperatures

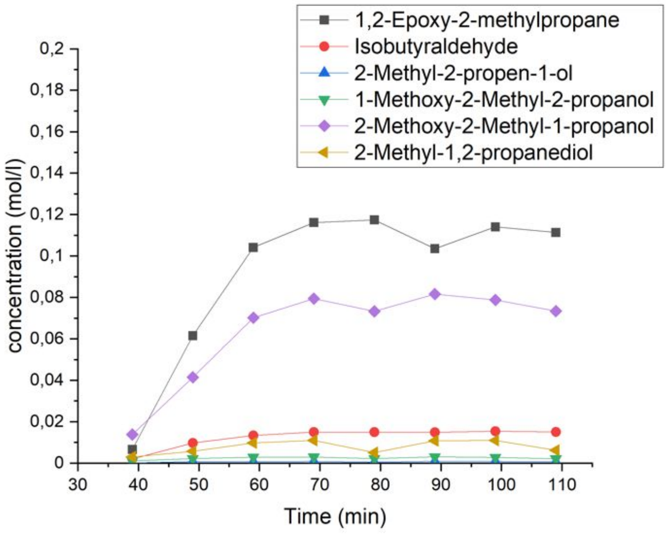

Figure S1. Concentration of the epoxide and the side products as a function of time at 25°C.

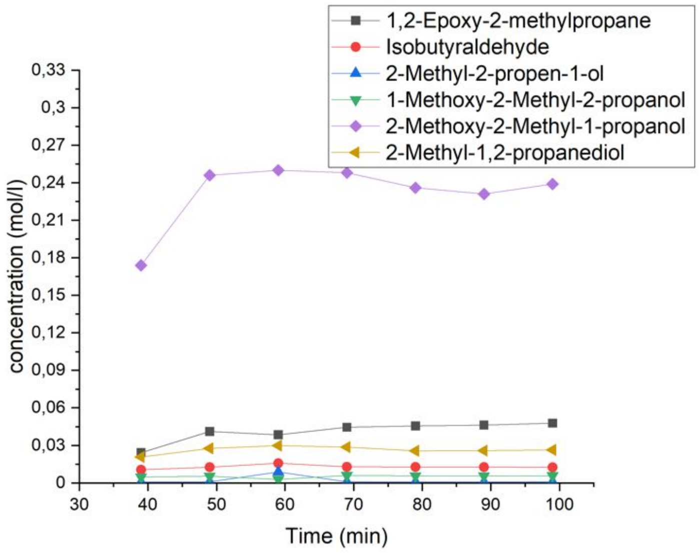

Figure S2. Concentration of the epoxide and the side products as a function of time at 30°C.

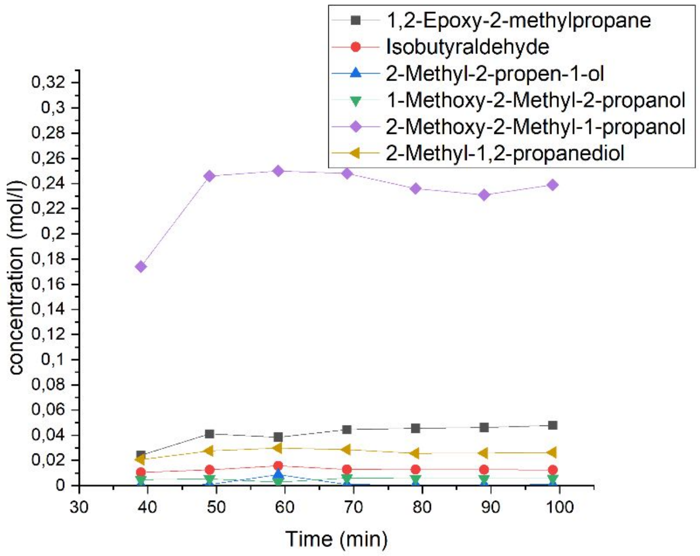

Figure S3. Concentration of the epoxide and the side products as a function of time at 40°C.

Dual plots of all by-products at various temperatures

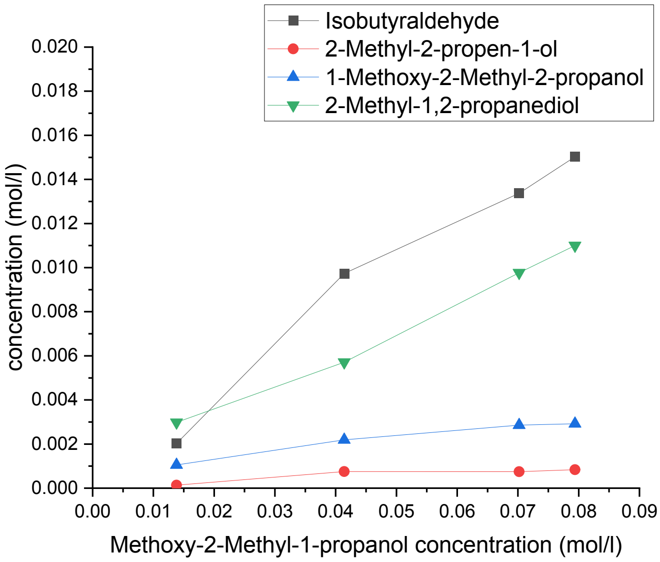

Figure S4. Concentration of the epoxide as a function of the concentrations of all side products at 25°C.

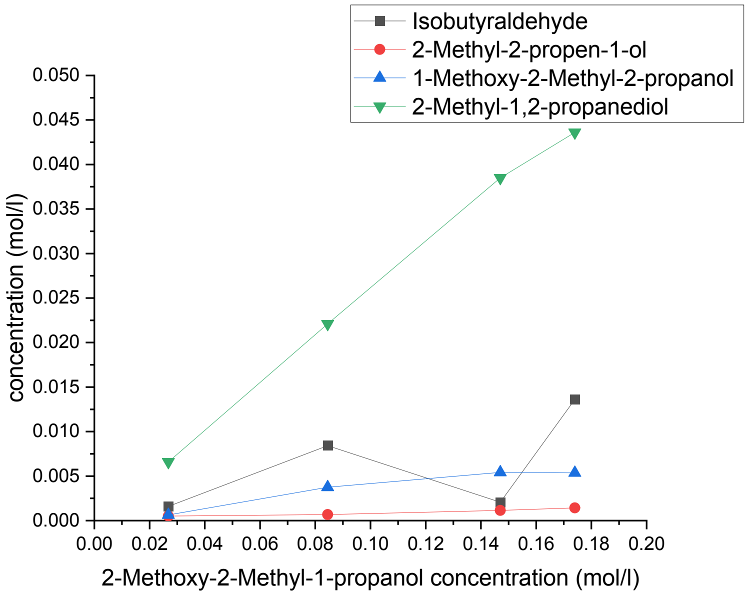

Figure S5. Concentration of the epoxide as a function of the concentrations of all side products at 30°C.

Table S2. Experimental matrix 1-butene

	Run	T (°C)	P (bar)	1-butene flow (mL/min)	H_2O_2 flow (mL/min)	H_2O_2 (wt%)	H_2O (wt%)	Methanol (wt%)
	Experimental set up							
A	1	45	1	5	0.5	2	5	93
	2	45	1	2.5	0.5	2	5	93
	3	45	1	7.5	0.5	2	5	93
	4	45	1	5	0.5	2	5	93
B	5	20	1	5	0.5	2	5	93
	6	30	1	5	0.5	2	5	93
	7	40	1	5	0.5	2	5	93
	8	50	1	5	0.5	2	5	93
	9	20	1	5	0.5	2	5	93
C	10	40	1	5	0.5	2	5	93
	11	40	1	5	0.5	2	20	78
	12	40	1	5	0.5	2	30	68
	13	40	1	5	0.5	2	40	58
	14	40	1	5	0.5	2	5	93
D	15	40	1	5	0.5	1	2	97
	16	40	1	5	0.5	2	5	93
	17	40	1	5	0.5	4	9	87
	18	40	1	5	0.5	1	2	97
E	19	40	1	5	0.5	2	5	93
	20	40	1	5	1	2	5	93
	21	40	1	5	2	2	5	93
	22	40	1	5	0.5	2	5	93

Table S3. Experimental matrix isobutene

	Run	T	P	Iso-butene flow	H_2O_2 flow	H_2O_2	H_2O	Methanol
		°C	bar	ml/min	ml/min	wt%	wt%	wt%
	Experimental setup							
A	1	15	1	9	0.5	2	5	93
	2	15	1	9	0.5	4	9	87
	3	15	1	9	0.5	5	11	84
	4	15	1	9	0.5	8	17	75
B	5	15	1	9	0.5	2	5	93
	6	15	1	9	0.5	2	20	78
	7	15	1	9	0.5	2	30	68
	8	15	1	9	0.5	2	40	58
C	9	15	1	9	0.5	2	5	93
	10	25	1	9	0.5	2	5	93
	11	30	1	9	0.5	2	5	93
	12	40	1	9	0.5	2	5	93
D	13	15	1	9	0.5	2	5	93
	14	15	1	9	1	2	5	93
	15	15	1	9	2	2	5	93
	16	15	1	9	3	2	5	93
E	17	15	1	3	0.5	2	5	93
	19	15	1	9	0.5	2	5	93
	20	15	1	12	0.5	2	5	93
F / 24h	21	15	1	9	0.5	2	5	93
	22	15	1	9	0.5	2	5	93
	23	15	1	9	0.5	2	5	93
